# The association of smoking on the increased risk of osteoporotic fracture: Results from a cross-sectional study and two-sample Mendelian randomization

**DOI:** 10.18332/tid/189485

**Published:** 2024-06-27

**Authors:** Min Fang, Zhi Xia, Xueyao Rong, Jian Xiao

**Affiliations:** 1 Hunan Provincial Key Laboratory of the Research and Development of Novel Pharmaceutical Preparations, Changsha Medical University, Changsha, China; 2 “The 14th Five-Year Plan” Application Characteristic Discipline of Hunan Province (Pharmaceutical Science) Changsha Medical University, Changsha, China; 3 Department of Oncology, Hunan Provincial People's Hospital-First Affiliated Hospital of Hunan Normal University, Hunan Normal University, Changsha, China; 4 Department of Geriatric Respiratory and Critical Care Medicine, Xiangya Hospital, Central South University, Changsha, China; 5 National Clinical Research Center for Geriatric Disorders, Xiangya Hospital, Central South University, Changsha, China

**Keywords:** smoking, osteoporosis, fracture, NHANES, Mendelian randomization

## Abstract

**INTRODUCTION:**

We conducted analyses of the association between smoking and osteoporosis and osteoporotic fractures using a secondary dataset analysis of the National Health and Nutrition Examination Survey (NHANES) database and the two-sample Mendelian randomization (MR) method.

**METHODS:**

The associations between smoking and osteoporosis or osteoporotic fractures were analyzed using weighted logistic regression models for both univariate and multivariable analyses using pooled 1999–2018 NHANES data. The summary-level data of genome-wide association studies (GWAS) of smoking and osteoporosis were extracted from the IEU Open GWAS project. The inverse variance weighted method was used as the main method for the two-sample MR analysis.

**RESULTS:**

We obtained the following main findings based on the NHANES data: smoking was associated with osteoporosis according to the analyses of 30856 participants (OR=1.21; 95% CI: 1.06–1.39, p=0.004); smoking was associated with hip osteoporotic fracture according to the analyses of 30928 participants (OR=1.47; 95% CI: 1.14–1.90, p=0.004); smoking was associated with wrist osteoporotic fracture according to the analyses of 30923 participants (OR=1.33; 95% CI: 1.18–1.49, p<0.001); and smoking was associated with spine osteoporotic fracture according to the analyses of 30910 participants (OR=1.43, 95% CI: 1.18–1.73, p<0.001). In addition, we confirmed the potential causal effect of smoking on the risk of osteoporotic fracture (OR=24.5; 95% CI: 1.11–539, p=0.043) by conducting two-sample MR analyses.

**CONCLUSIONS:**

Smoking was associated with increased risks of both osteoporosis and osteoporotic fracture. Smoking showed a potential causal effect on the risk of osteoporotic fracture.

## INTRODUCTION

Smoking is a global public health problem with widespread impacts, which is widely recognized as a causative risk factor for chronic obstructive pulmonary disease and lung cancer. In recent years, besides the diseases of the respiratory system, a growing number of studies have found that smoking is associated with various diseases, such as liver disease^1^, rheumatoid arthritis^2^, and multiple sclerosis^3^. Osteoporosis is a systemic skeletal disorder characterized by the reduction of bone mass, bone mineral density, and even changes in the structure of bone, which greatly increases the risk of fracture^4^. As previously documented, the prevalence of osteoporosis in developing and developed countries was 22.1% and 14.5%, respectively, and the global overall prevalence of osteoporosis was 19.7%^5^, indicating a high incidence of osteoporosis in human beings. Furthermore, fractures are identified as the most serious complication of osteoporosis, which causes heavy burdens for patients and society^6^.

Studies found that cigarette smoking increased the risk of osteoporosis in patients with inflammatory bowel diseases^7^. An Asian biobank reported that tobacco use had profound influences on osteoporosis^8^. In addition, a retrospective case-control study suggested that smoking was also a potential risk factor for osteoporosis in patients with non-small cell lung cancer^9^. Smoking has been shown to cause an imbalance in bone turnover, making bones susceptible to osteoporosis and fragility fractures^10^. Research also discovered that smoking exerted promoting effects on osteoporotic fractures in both males and females^11,12^. However, despite these past cognitions, given the prevalence of smoking and the dangers of osteoporotic fractures, the current understanding of the relationship between them is underwhelming.

In this study, we conducted in-depth analyses of the association between smoking and osteoporosis and osteoporotic fractures using the cross-sectional study of the National Health and Nutrition Examination Survey (NHANES) and the two-sample Mendelian randomization (MR) method.

## METHODS

### The NHANES

The National Center for Health Statistics (NCHS) of the Centers for Disease Control and Prevention (CDC) conducts the ongoing project of NHANES (https://www.cdc.gov/nchs/nhanes/index.htm), a US serial cross-sectional study focusing on a variety of health and nutrition measurements to be used to determine the prevalence of major diseases and risk factors for many diseases. The NCHS Research Ethics Review Board authorized the NHANES protocols, and at the time of recruitment, all individuals signed informed consent forms. No additional ethical approval was required as the data for our current study were obtained from the publicly available database. Five data sections of the NHANES – dietary, laboratory, examination, demographic, and questionnaire – are publicly accessible.

### Smoking status and osteoporosis relative data in NHANES

Survey data on smoking status were gathered via questionnaires among US adults. Participants were classified in the smoking group if they had smoked 100 cigarettes or more over their lifetime; otherwise, they were included in the non-smoking group^13^. Osteoporosis and osteoporotic fracture data were also gathered via questionnaires among US adults: ‘Has a doctor ever told you that you had osteoporosis?’, and ‘Has a doctor ever told you that you had broken or fractured your hip/wrist/spine?’.

In NHANES, from 1999 to 2018, smoking status data were shown in all survey cycles, while osteoporosis data were included in eight survey cycles (1999–2000, 2001–2002, 2003–2004, 2005–2006, 2007–2008, 2009–2010, 2013–2014, and 2017–2018) with two cycles deficient (2011–2012 and 2015–2016). Therefore, all data extraction in our current study came from these eight survey cycle datasets where osteoporosis was located.

### Demographic variables in NHANES

This study included the following demographic covariates: gender, age, race, education level, marital status, and the ratio of family income to poverty (PIR). Two age categories (≥65 and <65 years) were created. Mexican American, Other Hispanic, Non-Hispanic White, Non-Hispanic Black, and Other Race (including multi-racial) were the five categories under which races fall. Three education categories were established: lower than high school, high school or equivalent, and college or higher. The three marital status categories were married/living with a partner, widowed/divorced/separated, and never married. In addition, three groups of PIR were also created: ≤1.30, >1.30 to ≤3.50, and >3.50.

### Body mass index and calcium in NHANES

The covariate of body mass index (BMI) was extracted from the examination data and divided into three groups: <25, 25 to <30, and ≥30 (kg/m^2^). The total calcium concentration (mg/dL) covariate was extracted from the laboratory data and divided into two groups based on the median value (9.5 mg/dL).

### Statistical analysis for NHANES

NHANES statistical analyses were performed according to CDC guidelines (https://wwwn.cdc.gov/nchs/nhanes/tutorials/default.aspx). All the statistical analyses were performed using R software (version 4.3.0, https://www.r-project.org/) with the *survey* and *nhanesR* packages^14^. Statistical significance was considered for a two-tailed p<0.05.

The results of baseline characteristics for the included variables are presented as frequencies with percentages. The weighted chi-squared test was used to determine the difference in distribution between baseline characteristic groups. The association between smoking and osteoporosis was analyzed using weighted logistic regression models for univariate and multivariable analyses, and their odds ratios (ORs) with corresponding 95% confidence intervals (CIs) were calculated. For the univariate analyses (Model 0), no covariates were adjusted. The multivariable analysis models were as follows: Model 1 adjusted for age, gender, and race; Model 2 further adjusted for education level, marital status, and PIR; and Model 3 further adjusted for BMI and calcium. The main results were based on the primary analyses, in which we removed the missing values for all included variables. Meanwhile, to verify the stability of our study, we conducted sensitivity analyses to classify the missing values of the covariates as ‘not recorded’ groups instead of removing them. Stratified analyses based on age, gender, race, education level, marital status, PIR, BMI, and calcium were also performed.

### Mendelian randomization (MR) study design

MR analyses relied on three assumptions (Supplementary file Figure S1): 1) that the instrumental variable (IV) is closely associated with the exposure; 2) that the IV is not associated with confounders; and 3) that the IV can only influence the outcome through the exposure and not in other ways^15^. In our current study, two-sample MR analyses were used to explore the causal association between smoking and osteoporosis. However, according to the definition requirements of the two-sample MR, the two samples should come from the same population but should not involve overlapping participants^15^.

### Smoking and osteoporosis data sources for MR

We searched the summary-level data of genomewide association studies (GWAS) of smoking and osteoporosis in the IEU Open GWAS project (https://gwas.mrcieu.ac.uk), a database of more than 346 billion genetic associations from over 50 thousand GWAS summary datasets for querying or download. By using the keywords ‘smoking’ and ‘osteoporosis’, we searched and selected the appropriate GWAS summary datasets from the search results of both for MR analyses. No additional ethical approval was required as the data for our study were obtained from publicly available databases.

### IV selection for MR

We screened the single nucleotide polymorphism (SNPs) as IVs through the following steps. First, the association of exposure threshold was set at p<5×10^-8^, and the effect of linkage disequilibrium (LD) was excluded (r^2^<0.001 within a distance of 10000 kb). Second, F-statistics (F=β^2^/SE^2^) were calculated for the SNPs to measure the strength of the instruments, and an F-statistic <10 was excluded because of the ‘weak instrument’^16^. Third, SNPs that were associated with the outcome (p<5×10^-8^) were excluded. Fourth, proxies were identified in high LDs (r^2^ >0.8) for those SNPs that were absent in the outcome, while those absent SNPs for which no suitable proxies were identified were excluded. Fifth, SNPs with minor allele frequency (MAF) <0.01 were excluded. Sixth, harmonization was performed to align the alleles of exposure and outcome SNPs, and SNPs were removed for being palindromic with intermediate allele frequencies. Seventh, the selected SNPs were manually searched using the PhenoScanner (http://www.phenoscanner.medschl.cam.ac.uk), a database of publicly available results from large-scale human genetic association studies in which genetic variants are cross-referenced for association with many phenotypes of different types^17^, to exclude SNPs affected by known confounders.

### Statistical analyses for MR

Inverse variance weighted (IVW) is the main method commonly used in MR analysis, which combines all Wald ratios for each SNP to obtain a pooled estimate^15^. Therefore, the random effects IVW method was performed as the primary method in this study. Under the InSIDE assumption, performing a weighted linear regression of the outcome coefficients on the exposure coefficients^18^, the MR-Egger regression was used as the complementary analysis. Additionally, weighted median, simple mode, and weighted mode were used as complementary analyses. Leave-one-out analysis was used to evaluate whether the MR estimate was driven or biased by a single SNP^15^. The IVW and MR-Egger methods were used to test for heterogeneity. Cochran’s Q statistic and p-value were used to determine the presence of heterogeneity, with p<0.05 indicating the presence of heterogeneity. The MR pleiotropy residual sum and outlier (MR-PRESSO) global test and the MR-Egger intercept test were used to monitor the horizontal pleiotropy effect, and the evidence of pleiotropy was p<0.05^19^. All the MR analyses were performed with the R packages of *TwoSampleMR*, and MR-PRESSO using R software. A two-tailed p<0.05 was considered statistically significant.

## RESULTS

### The association between smoking and osteoporosis based on NHANES

A total of 81589 potential participants were extracted from the NHANES (1999–2000, n=9965; 2001–2002, n=11039; 2003–2004, n=10122; 2005–2006, n=10348; 2007–2008, n=10149; 2009–2010, n=10537; 2013–2014, n=10175; and 2017–2018, n=9254). By removing the missing data of the included variables, 30856 participants remained to be used for primary analyses, and their baseline characteristics are given in the Supplementary file Table S1. The distributions of age, gender, race, education level, marital status, PIR, and BMI were statistically different between those with osteoporosis.

Though the univariate analysis result (Model 0) showed no obvious association between smoking and osteoporosis, multivariable analysis results (Model 1 to 3: OR=1.28; 95% CI: 1.12–1.46, p<0.001; OR=1.22; 95% CI: 1.07–1.39, p=0.003; OR=1.21; 95% CI: 1.06–1.39, p=0.004, respectively) have displayed the definite associations between them after adjusting the covariates of age, gender, race, education level, marital status, PIR, BMI, and calcium (Table 1). Meanwhile, in the sensitivity analyses that included 37214 individuals (Supplementary file Table S2), we found similar results and trends (Table 1). Further detailed analysis results of the primary and sensitivity analyses are presented in the Supplementary file Tables S3–S6.

**Table 1:** The associations of smoking to osteoporosis in different analysis models among US adults who responded to smoking status and osteoporosis questionnaires in the National Health and Nutrition Examination Survey (NHANES), data from 1999–2010, 2013–2014, and 2017–2018 (N=30856 for primary analysis; N=37214 for sensitivity analysis)

*Model*	*Smoking*	*Primary results of osteoporosis*		*Sensitivity results of osteoporosis*	
		*OR (95% CI)*	*p*	*OR (95% CI)*	*p*
Model 0	No ®	1		1	
	Yes	1.00 (0.88–1.13)	0.972	1.00 (0.89–1.12)	0.984
Model 1	No ®	1		1	
	Yes	1.28 (1.12–1.46)	<0.001	1.28 (1.14–1.44)	<0.001
Model 2	No ®	1		1	
	Yes	1.22 (1.07–1.39)	0.003	1.23 (1.09–1.38)	<0.001
Model 3	No ®	1		1	
	Yes	1.21 (1.06–1.39)	0.004	1.22 (1.08–1.37)	0.001

Model 0: univariate analysis. Model 1: multivariable analysis adjusted for age, gender, and race. Model 2: multivariable analysis adjusted for the variables in Model 1 and also including education level, marital status, and the ratio of family income to poverty. Model 3: multivariable analysis adjusted for the variables in Model 2 and also including body mass index and calcium.

® Reference categories.

**Figure 1:**
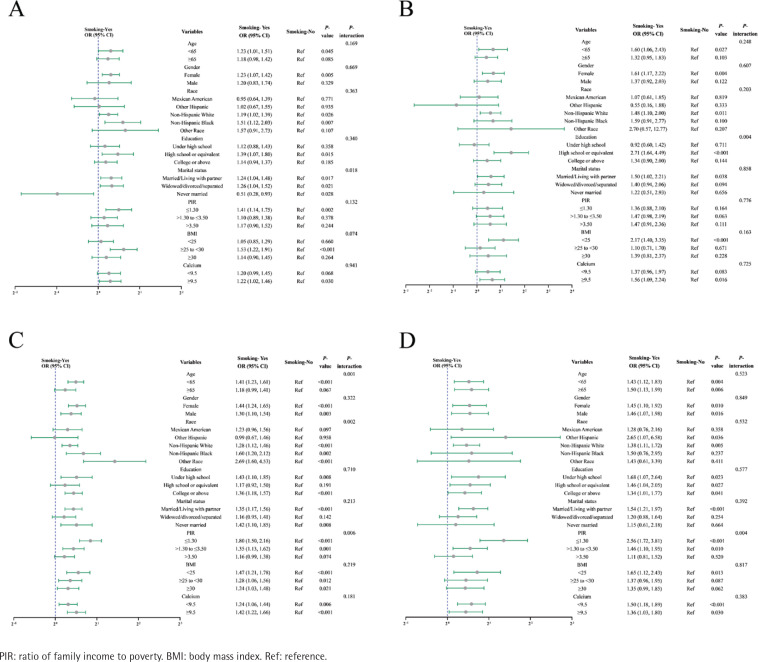
Forest plots of stratified analyses for the correlative relationship of smoking with osteoporosis (A), hip osteoporotic fracture (B), wrist osteoporotic fracture (C), and spine osteoporotic fracture (D) among US adults who responded to smoking status, osteoporosis, and osteoporotic fracture questionnaires in the National Health and Nutrition Examination Survey (NHANES), data from 1999–2010, 2013–2014, and 2017–2018 (N=30856, N=30928, N=30923, and N=30910 for stratified analyses on the osteoporosis, hip osteoporotic fracture, wrist osteoporotic fracture, and spine osteoporotic fracture respectively)

In the stratified analyses, based on the included covariates of age, gender, race, education level, marital status, PIR, BMI, and calcium, we performed multivariable analysis by adjusting all these covariates except the stratified target covariate itself. Results indicated that there was an interaction between smoking and marital status (p-interaction=0.018) in the relationship of smoking with osteoporosis (Figure 1A). We found that smoking showed a potential protective effect for osteoporosis in the ‘never married’ subgroup (OR=0.51; 95% CI: 0.28–0.93, p=0.028) (Figure 1A).

### The association between smoking and osteoporotic fractures based on NHANES

There were 30928 and 37298 participants used to conduct primary and sensitivity analyses, respectively, on the association between smoking and hip osteoporotic fracture (Supplementary file Tables S7 and S8). Both univariate and multivariable analysis results showed associations between smoking and hip osteoporotic fracture (Model 0 to 3: OR=1.65; 95% CI: 1.28–2.11, p<0.001; OR 1.60, 95% CI: 1.24–2.08, p<0.001; OR=1.47; 95% CI: 1.14–1.91, p=0.004; OR=1.47; 95% CI: 1.14–1.90, p=0.004, respectively) (Table 2 and Supplementary file Tables S9–S12). Similar results and trends were found in the sensitivity analyses (Table 2 and Supplementary file Tables S9–S12).

**Table 2:** The associations of smoking to osteoporotic fractures in different analysis models among US adults who responded to smoking status and osteoporotic fracture questionnaires in the National Health and Nutrition Examination Survey (NHANES), data from 1999–2010, 2013–2014, and 2017–2018 (N=30928, N=30923, and N=30910 for primary analyses on the hip, wrist, and spine osteoporotic fractures, respectively; N=37298, N=37297, and N=37280 for sensitivity analyses on the hip, wrist, and spine osteoporotic fractures, respectively)

	*Model*	*Smoking*	*Primary results*		*Sensitivity results*	
			*OR (95% CI)*	*p*	*OR (95% CI)*	*p*
Hip	Model 0	No ®	1		1	
		Yes	1.65 (1.28–2.11)	<0.001	1.53 (1.22–1.92)	<0.001
	Model 1	No ®	1		1	
		Yes	1.60 (1.24–2.08)	<0.001	1.51 (1.19–1.91)	<0.001
	Model 2	No ®	1		1	
		Yes	1.47 (1.14–1.91)	0.004	1.39 (1.09–1.77)	0.007
	Model 3	No ®	1		1	
		Yes	1.47 (1.14–1.90)	0.004	1.39 (1.10–1.76)	0.007
Wrist	Model 0	No ®	1		1	
		Yes	1.44 (1.30–1.61)	<0.001	1.51 (1.36–1.67)	<0.001
	Model 1	No ®	1		1	
		Yes	1.32 (1.18–1.49)	<0.001	1.37 (1.23–1.53)	<0.001
	Model 2	No ®	1		1	
		Yes	1.33 (1.18–1.49)	<0.001	1.38 (1.23–1.54)	<0.001
	Model 3	No ®	1		1	
		Yes	1.33 (1.18–1.49)	<0.001	1.38 (1.23–1.54)	<0.001
Spine	Model 0	No ®	1		1	
		Yes	1.60 (1.32–1.94)	<0.001	1.64 (1.37–1.96)	<0.001
	Model 1	No ®	1		1	
		Yes	1.47 (1.21–1.78)	<0.001	1.50 (1.25–1.80)	<0.001
	Model 2	No ®	1		1	
		Yes	1.42 (1.17–1.72)	<0.001	1.44 (1.20–1.73)	<0.001
	Model 3	No ®	1		1	
		Yes	1.43 (1.18–1.73)	<0.001	1.46 (1.21–1.75)	<0.001

Model 0: univariate analysis. Model 1: multivariable analysis adjusted for age, gender, and race. Model 2: multivariable analysis adjusted for the variables in Model 1 and also including education level, marital status, and the ratio of family income to poverty. Model 3: multivariable analysis adjusted for the variables in Model 2 and also including body mass index and calcium.

® Reference categories.

In addition, 30923 and 37297 participants were applied to conducting primary and sensitivity analyses on the association between smoking and wrist osteoporotic fracture (Supplementary file Tables S13 and S14). Results from univariate and multivariable analysis established the relationships between smoking and wrist osteoporotic fracture (Model 0 to 3: OR=1.44; 95% CI: 1.30–1.61, p<0.001; OR=1.32, 95% CI: 1.18–1.49, p<0.001; OR=1.33, 95% CI: 1.18–1.49, p<0.001; OR=1.33, 95% CI: 1.18–1.49, p<0.001, respectively) (Table 2 and Supplementary file Tables S15–S18). Similar results and trends were observed in the sensitivity analyses (Table 2 and Supplementary file Table S15–S18).

Furthermore, primary and sensitivity analyses were conducted to explore the association between smoking and spine osteoporotic fracture involving 30910 and 37280 participants, respectively (Supplementary file Tables S19 and S20). As expected, both univariate and multivariable analysis results exhibited associations between smoking and spine osteoporotic fracture (Model 0 to 3: OR=1.60; 95% CI: 1.32–1.94, p<0.001; OR=1.47; 95% CI: 1.21–1.78, p<0.001; OR=1.42; 95% CI: 1.17–1.72, p<0.001; OR=1.43; 95% CI: 1.18–1.73, p<0.001, respectively) (Table 2 and Supplementary file Tables S21–S24). Similar results and trends were found in the sensitivity analyses (Table 2 and Supplementary file Tables S21–S24).

According to the results of stratified analyses, we found that there was: an interaction between smoking and education (p-interaction=0.004) in the correlative relationship of smoking with hip osteoporotic fracture, which the subgroup of ‘high school or equivalent’ displayed relatively obvious association (OR=2.71; 95% CI: 1.64–4.49, p<0.001) (Figure 1B); interactions between smoking and age, race, or PIR (p-interaction=0.001, p-interaction=0.002, p-interaction=0.006, respectively) in the correlative relationship of smoking with wrist osteoporotic fracture, which the subgroups of age <65 years (OR=1.41; 95% CI: 1.23–1.61, p<0.001), Other race (OR=2.69; 95% CI: 1.60–4.53, p<0.001), as well as PIR≤1.30 (OR=1.80; 95% CI: 1.50–2.16, p<0.001) displayed relatively obvious associations (Figure 1C); and an interaction between smoking and PIR (p-interaction=0.004) in the correlative relationship of smoking with spine osteoporotic fracture, which the subgroup of PIR≤1.30 displayed relatively obvious association (OR=2.56; 95% CI: 1.72–3.81, p<0.001) (Figure 1D).

### Two-sample MR analyses indicated the causality of smoking on osteoporotic fracture

We screened out six smoking (ukb-a-16, ukb-a-17, ukb-a-224, ukb-a-225, ukb-b-223, and ukb-b-2134), one osteoporosis (finn-b-M13_OSTEOPOROSIS), and one osteoporotic fracture (finn-b-OSTEOPOROSIS_FRACTURE_FG) GWAS summary datasets from the IEU Open GWAS project (Supplementary file Table S25). IVW method was used to preliminary explore the causal association between smoking and osteoporosis or osteoporotic fracture. The vast majority of the analysis results showed the trend for smoking to cause osteoporosis but did not reflect statistical significance (Supplementary file Table S26). The results also displayed the trend for smoking to cause osteoporotic fracture, and the ukb-a-16 showed statistical significance among them (Supplementary file Table S26).

In detail, according to the SNPs selection process, we screened out 16 IVs for the two-sample MR analysis of ukb-a-16 (exposure: smoking) and finn-b-OSTEOPOROSIS_FRACTURE_FG (outcome: osteoporotic fracture) (Supplementary file Table S27). The F-statistics corresponding to the single SNPs ranged from 30 to 103, suggesting that the causal association was unlikely to be affected by weak IV bias. MR analysis result of IVW indicated the causal effect of smoking on the risk of osteoporotic fracture (OR=24.5; 95% CI: 1.11–539, p=0.043) (Table 3). Results of other methods of MR-Egger, weighted median, simple mode, and weighted mode showed the same trend with IVW, though without statistical significance (Table 3 and Supplementary file Figures S2 and S3). In leave-one-out analyses, results indicated that no single SNP strongly drove the overall effect of smoking on osteoporotic fracture (Supplementary file Figure S4).

**Table 3:** Two-sample Mendelian randomization (MR) results of the causal effect of smoking on osteoporotic fracture based on the summary-level data of genome-wide association studies (GWAS) datasets from the IEU Open GWAS database

*Exposure (smoking)*	*Outcome (Osteoporotic fracture)*	*Methods*	*IVs*	*OR (95% CI)*	*p*	*Heterogeneity p*		*Pleiotropy p*	
						*IVW*	*MR-Egger*	*MR-PRESSO*	*MR-Egger*
ukb-a-16	finn-b-OSTEOPOROSIS_FRACTURE_FG	MR-Egger	16	3.14×10^4^ (0.0150–6.55×10^10^)	0.185	0.745	0.753	0.776	0.341
		Weighted median	16	8.63 (0.115–647)	0.328				
		IVW	16	24.5 (1.11–539)	0.043				
		Simple mode	16	2.10 (0.00101–4390)	0.851				
		Weighted mode	16	2.88 (0.00215–3840)	0.777				

IV: instrumental variable. MR: Mendelian randomization. IVW: inverse variance weighted.

The heterogeneity was assessed using IVW and MR-Egger methods, with results indicating no intergenic heterogeneity in SNPs as both had p>0.05 (Table 3). In addition, the MR-PRESSO global test and the MR-Egger intercept test results indicated that the possibility of pleiotropy was weak (p>0.05) (Table 3).

## DISCUSSION

Smoking-induced osteoporosis and osteoporotic fracture should be given more attention. In our current study, using a pooled analysis of the NHANES database, we discovered the association between smoking and the increased risk of osteoporosis after adjusting the covariates of age, gender, race, education level, marital status, PIR, BMI, and calcium. Both univariate and multivariable analysis models found associations between smoking and the increased risks of hip, wrist, as well as spine osteoporotic fractures. Similar tendencies were shown in the findings of the primary and sensitivity analyses. Moreover, we conducted the two-sample MR analysis and confirmed the potential causal effect of smoking on the risk of osteoporotic fracture.

By including 4226 US adults with complete information on smoking history from the 2005–2010 NHANES, Thompson et al.^20^ concluded that women who smoked for more than 30 pack-years had twice the prevalence of osteoporosis compared to women who had never smoked, while there was no association between men’s smoking history and osteoporosis. Our study included 30856 or more participants and found that both males and females had the same association trends between smoking and osteoporosis. In contrast, according to the stratified analysis, the males showed no statistical significance. Hou et al.^21^ examined the NHANES data from 2005 to 2010, 2013 to 2014, and 2017 to 2018 and obtained 10564 individuals defined as osteoporosis or osteopenia based on bone mineral density, concluding that smoking was associated with a higher prevalence of osteoporosis than non-smoking. Correspondingly, we reached a similar conclusion based on a much larger osteoporosis sample based on the questionnaire.

Previous studies have mostly been concerned and reported that smoking increases the risk of hip fracture^11,12^. However, the osteoporosis questionnaires in NHANES focus not only on hip fractures but also on wrist and spine fractures, which are common complications of osteoporosis^22,23^. In our current study, we performed a comprehensive study of smoking and osteoporotic fractures of the hip, wrist, and spine using the NHANES database, indicating that smoking raised the risk of these types of fractures.

In recent years, it has been found that smoking can lead to osteoporosis through different mechanisms. Jing et al.^24^ reported that tobacco toxins induced osteoporosis through ferroptosis in rat bone marrow mesenchymal stem cells by accumulating intracellular reactive oxygen species activated AMPK signaling. Li et al.^25^ discovered that the risk of osteoporosis was partly mediated by cadmium from smoking, according to the study of the Swedish cohort of Osteoporotic Fractures. By using machine learning algorithms, Wang et al.^26^ concluded that smoking-related osteoporosis and chronic obstructive pulmonary disease shared pathogenesis in which immune cell infiltration profiles play a significant role. Smoking also impacted the RANK-RANKL-OPG pathway and intestinal microbiota composition, directly and indirectly affecting bone mineral density^7^. As tobacco smoke contains more than 7000 chemical compounds^7^, the mechanisms of smoking-induced osteoporosis still need further research.

Race contributes to the differences in the prevalence of osteoporosis, falls, and fractures^27^. Osteoporosis was reported to be more prevalent among those who were less educated and had lower incomes^28^. Higher income and education were associated with better awareness and knowledge about osteoporosis^29^. Individuals who sought assessment for osteoporosis were older and more likely to be married than those who did not seek assessment^30^. Not only were these above factors as covariates needed to be considered in this study, but also their interactions with smoking in the association between smoking and osteoporosis/ osteoporotic fracture should be taken into account. Specifically, our stratified analyses revealed interactions between smoking and marital status in the association of smoking with osteoporosis; smoking and education in the association of smoking with hip osteoporotic fracture; smoking and age, race, or PIR in the association of smoking with wrist osteoporotic fracture; and smoking and PIR in the association of smoking with spine osteoporotic fracture, indicating that the relationship of smoking to the risk of developing osteoporosis and osteoporotic fracture is influenced by some other factors.

MR is an analytic method for evaluating the causality of an observed relationship between a risk factor or modifiable exposure and a clinically relevant outcome^31^. It plays an important role in the study of numerous diseases^32,33^. MR also provides new perspectives for research in the field of osteoporosis^34^. However, no MR has been previously applied to study the causal relationship between smoking and osteoporosis. In this study, though we did not find a statistically significant association between smoking and osteoporosis, the causal effect of smoking on the risk of osteoporotic fracture has been found by using the two-sample MR analysis.

### Strengths and limitations

This study has several strengths. By using the widely known US NHANES data, we included the sample sizes of more than 30000 participants to obtain reliable analysis results. Hereafter, we comprehensively investigated the association between smoking and osteoporotic fractures according to the NHANES data. In addition, using the modularized European GWAS summary datasets also with larger sample sizes, we further studied the causal effect of smoking on the risk of osteoporosis and osteoporotic fracture based on two-sample MR analyses. Our findings not only affirmed that smoking was a risk factor for osteoporosis but also emphasized its potential causal relationship with osteoporotic fracture. As previous studies described, exposure to secondhand smoke was also positively associated with osteoporosis^35,36^. We, therefore, advocate quitting smoking to eliminate the harm of smoking to osteoporosis.

Our study still has some limitations. First, two cycles (2011–2012 and 2015–2016) of osteoporosis data were deficient in NHANES, resulting in broken coherence in data collection. Second, participants with missing records from the NHANES (mainly for smoking and osteoporosis) were excluded from the present study and, therefore, may not represent the real-world situation. Third, NHANES is a cross-sectional survey rather than a prospective study, so this study cannot obtain hazard ratios for the associations of smoking with the risk of osteoporosis and osteoporotic fracture incidence. Fourth, the data for NHANES were from the American population that targeted the non-institutionalized civilian residents, and the MR data used in our current research were from European populations, which may have implications for the generalizability of our findings to other populations. Fifth, as with all MR studies, we could not address the issue of unobserved pleiotropy. Sixth, although the causal trends obtained by the five MR analysis methods were consistent, only IVW showed a statistically significant association with osteoporotic fracture that down-toned our argument. Finally, the 95% CIs of MR results of the causal effect of smoking on osteoporotic fracture showed large spans; GWAS studies in the future with larger sample sizes are needed to verify the stability of the MR results.

## CONCLUSIONS

Smoking is associated with the increased risks of osteoporosis, hip osteoporotic fracture, wrist osteoporotic fracture, and spine osteoporotic fracture based on NHANES data, and smoking shows potential causality for osteoporotic fracture, according to MR analysis. To the best of our knowledge, this is the first two-sample MR study to investigate the relationship between smoking and osteoporosis or osteoporotic fracture. However, further studies, such as randomized clinical trials with longer follow-ups, are warranted to provide more robust evidence.

## Supplementary Material



## Data Availability

The datasets generated and analyzed in this study are available at the NHANES website: https://www.cdc.gov/nchs/nhanes/index.htm. The GWAS summary data are available at https://gwas.mrcieu.ac.uk/.
