# Supplementary material for: The association of smoking on the increased risk of osteoporotic fracture: Results from a cross-sectional study and two-sample Mendelian randomization
Source: Tob Induc Dis. 2024 Jun 27;22:10.18332/tid/189485. doi: 10.18332/tid/189485 (PMC11201227; doi:10.18332/tid/189485)
Supplement: Supplementary file 1 [file TID-22-119-s1.pdf]

The association of smoking on the increased risk of osteoporotic fracture: results from a cross-sectional study and two-sample Mendelian randomization

Supplementary Tables and Figures

Table S1 Baseline characteristics of participants by osteoporosis for the primary analysis among US adults, who responded to smoking status and osteoporosis questionnaires in the National Health and Nutrition Examination Survey (NHANES), data from 1999-2010, 2013–2014, and 2017–2018 (N=30856)

**Variable**	**Total (N=30856)**	**Osteoporosis-No (N=28887)**	**Osteoporosis-Yes (N=1969)**	***P*-value**
Age, N (%)				< 0.001
<65	22512 (73.0)	21804 (82.9)	708 (43.1)	
≥65	8344 (27.0)	7083 (17.1)	1261 (56.9)	
Gender, N (%)				< 0.001
Female	15921 (51.6)	14244 (49.6)	1677 (87.2)	
Male	14935 (48.4)	14643 (50.4)	292 (12.8)	
Race, N (%)				< 0.001
Mexican American	5755 (18.7)	5516 (7.7)	239 (3.4)	
Other Hispanic	2098 (6.8)	1986 (4.9)	112 (3.2)	
Non-Hispanic White	15236 (49.4)	13964 (71.2)	1272 (83.0)	
Non-Hispanic Black	5916 (19.2)	5697 (10.5)	219 (5.4)	
Other Race	1851 (6.0)	1724 (5.8)	127 (5.0)	
Education, N (%)				< 0.001
Under high school	8678 (28.1)	8116 (17.7)	562 (20.8)	
High school or equivalent	7288 (23.6)	6751 (24.7)	537 (29.3)	
College or above	14890 (48.3)	14020 (57.6)	870 (49.9)	
Marital status, N (%)				< 0.001
Married/Living with partner	19153 (62.1)	18167 (66.1)	986 (56.3)	
Widowed/divorced/separated	7251 (23.5)	6360 (18.6)	891 (39.5)	
Never married	4452 (14.4)	4360 (15.3)	92 (4.2)	
PIR, N (%)				< 0.001
≤1.30	9054 (29.3)	8449 (19.8)	605 (22.5)	
>1.30 to ≤3.50	11864 (38.4)	11037 (35.6)	827 (42.1)	
>3.50	9938 (32.2)	9401 (44.5)	537 (35.4)	
BMI, N (%)				< 0.001
<25	9047 (29.3)	8367 (31.0)	680 (36.6)	
≥25 to <30	10785 (35.0)	10125 (34.3)	660 (31.6)	
≥30	11024 (35.7)	10395 (34.7)	629 (31.8)	
Calcium, N (%)				0.364
<9.5	16234 (52.6)	15250 (50.7)	984 (49.3)	
≥9.5	14622 (47.4)	13637 (49.3)	985 (50.7)	
Smoking, N (%)				0.972

Variable	Total (N=30856)	Osteoporosis-No (N=28887)	Osteoporosis-Yes (N=1969)	*P*-value
No	16107 (52.2)	15054 (52.1)	1053 (52.1)	
Yes	14749 (47.8)	13833 (47.9)	916 (47.9)	

PIR: ratio of family income to poverty; BMI: body mass index.

Table S2 Baseline characteristics of participants by osteoporosis for sensitivity analysis among US adults, who responded to smoking status and osteoporosis questionnaires in the National Health and Nutrition Examination Survey (NHANES), data from 1999-2010, 2013–2014, and 2017–2018 (N=37214)

**Variable**	**Total (N=37214)**	**Osteoporosis-No (N=34798)**	**Osteoporosis-Yes (N=2416)**	***P*-value**
Age, N (%)				< 0.001
<65	26776 (72.0)	25927 (82.3)	849 (41.7)	
≥65	10438 (28.0)	8871 (17.7)	1567 (58.3)	
Gender, N (%)				< 0.001
Female	19340 (52.0)	17267 (49.9)	2073 (87.5)	
Male	17874 (48.0)	17531 (50.1)	343 (12.5)	
Race, N (%)				< 0.001
Mexican American	7046 (18.9)	6739 (7.8)	307 (3.6)	
Other Hispanic	2644 (7.1)	2478 (5.1)	166 (3.9)	
Non-Hispanic White	17726 (47.6)	16218 (69.7)	1508 (81.9)	
Non-Hispanic Black	7544 (20.3)	7260 (11.4)	284 (5.8)	
Other Race	2254 (6.1)	2103 (6.0)	151 (4.9)	
Education, N (%)				< 0.001
Under high school	10951 (29.4)	10236 (18.7)	715 (21.4)	
High school or equivalent	8843 (23.8)	8204 (24.9)	639 (28.7)	
College or above	17362 (46.7)	16306 (56.3)	1056 (49.7)	
Not recorded	58 (0.2)	52 (0.1)	6 (0.2)	
Marital status, N (%)				< 0.001
Married/Living with partner	22398 (60.2)	21234 (64.1)	1164 (54.3)	
Widowed/divorced/separated	8837 (23.7)	7721 (18.5)	1116 (40.4)	
Never married	5484 (14.7)	5363 (15.8)	121 (4.6)	
Not recorded	495 (1.3)	480 (1.6)	15 (0.8)	
PIR, N (%)				< 0.001
≤1.30	10064 (27.0)	9364 (18.7)	700 (22.0)	
>1.30 to ≤3.50	13142 (35.3)	12217 (33.3)	925 (39.1)	
>3.50	10727 (28.8)	10155 (40.6)	572 (31.0)	
Not recorded	3281 (8.8)	3062 (7.3)	219 (7.9)	
BMI, N (%)				< 0.001
<25	10746 (28.9)	9935 (30.8)	811 (36.0)	
≥25 to <30	12666 (34.0)	11897 (33.5)	769 (29.7)	
≥30	12932 (34.8)	12190 (33.8)	742 (30.8)	
Not recorded	870 (2.3)	776 (1.8)	94 (3.5)	

Variable	Total (N=37214)	Osteoporosis-No (N=34798)	Osteoporosis-Yes (N=2416)	***P*-value**
Calcium, N (%)				0.506
<9.5	18564 (49.9)	17423 (48.5)	1141 (46.9)	
≥9.5	16264 (43.7)	15153 (46.0)	1111 (47.4)	
Not recorded	2386 (6.4)	2222 (5.5)	164 (5.7)	
Smoking, N (%)				0.984
No	19559 (52.6)	18252 (52.2)	1307 (52.2)	
Yes	17655 (47.4)	16546 (47.8)	1109 (47.8)	

PIR: ratio of family income to poverty; BMI: body mass index.

Table S3 Associations of all the included variables with osteoporosis based on model 0 among US adults, who responded to smoking status and osteoporosis questionnaires in the National Health and Nutrition Examination Survey (NHANES), data from 1999-2010, 2013–2014, and 2017–2018 (N=30856 for primary analysis; N=37214 for sensitivity analysis)

**Variable**	**Primary results**		**Sensitivity results**	
	**OR (95% CI)**	***P*-value**	**OR (95% CI)**	***P*-value**
Age				
<65	Ref	Ref	Ref	Ref
≥65	6.42 (5.68, 7.25)	<0.001	6.50 (5.83, 7.26)	<0.001
Gender				
Female	Ref	Ref	Ref	Ref
Male	0.14 (0.12, 0.17)	<0.001	0.14 (0.12, 0.17)	<0.001
Race				
Mexican American	Ref	Ref	Ref	Ref
Other Hispanic	1.47 (1.02, 2.11)	0.039	1.65 (1.20, 2.26)	0.002
Non-Hispanic White	2.60 (2.05, 3.29)	<0.001	2.57 (2.05, 3.21)	<0.001
Non-Hispanic Black	1.15 (0.86, 1.54)	0.347	1.11 (0.84, 1.46)	0.453
Other Race	1.91 (1.35, 2.72)	<0.001	1.78 (1.29, 2.44)	<0.001
Education				
Under high school	Ref	Ref	Ref	Ref
High school or equivalent	1.02 (0.84, 1.22)	0.868	1.01 (0.85, 1.19)	0.949
College or above	0.74 (0.63, 0.87)	<0.001	0.77 (0.66, 0.90)	0.001
Not recorded			1.49 (0.55, 4.07)	0.431
Marital status				
Married/Living with partner	Ref	Ref	Ref	Ref
Widowed/divorced/separated	2.50 (2.22, 2.81)	<0.001	2.58 (2.29, 2.89)	<0.001
Never married	0.32 (0.25, 0.41)	<0.001	0.34 (0.27, 0.43)	<0.001
Not recorded			0.57 (0.24, 1.40)	0.219
PIR				
≤1.30	Ref	Ref	Ref	Ref
>1.30 to ≤3.50	1.04 (0.89, 1.22)	0.601	1.00 (0.86, 1.16)	0.982
>3.50	0.70 (0.58, 0.85)	<0.001	0.65 (0.54, 0.78)	<0.001
Not recorded			0.92 (0.73, 1.17)	0.500
BMI				
<25	Ref	Ref	Ref	Ref
≥25 to <30	0.78 (0.68, 0.90)	<0.001	0.76 (0.67, 0.86)	<0.001
≥30	0.78 (0.67, 0.91)	0.001	0.78 (0.68, 0.89)	<0.001

Variable	Primary results		Sensitivity results	
	OR (95% CI)	*P*-value	OR (95% CI)	*P*-value
Not recorded			1.68 (1.27, 2.23)	<0.001
Calcium				
<9.5	Ref	Ref	Ref	Ref
≥9.5	1.05 (0.94, 1.18)	0.364	1.06 (0.95, 1.19)	0.268
Not recorded			1.08 (0.85, 1.36)	0.538
Smoking				
No	Ref	Ref	Ref	Ref
Yes	1.00 (0.88, 1.13)	0.972	1.00 (0.89, 1.12)	0.984

Model 0: univariate analysis; PIR: ratio of family income to poverty; BMI: body mass index; OR: odds ratio; CI: confidence interval; Ref: reference.

Table S4 Associations of smoking and other variables with osteoporosis based on model 1 among US adults, who responded to smoking status and osteoporosis questionnaires in the National Health and Nutrition Examination Survey (NHANES), data from 1999-2010, 2013–2014, and 2017–2018 (N=30856 for primary analysis; N=37214 for sensitivity analysis)

**Variable**	**Primary results**		**Sensitivity results**	
	**OR (95% CI)**	***P*-value**	**OR (95% CI)**	***P*-value**
Age				
<65	Ref	Ref	Ref	Ref
≥65	6.29 (5.52, 7.16)	<0.001	6.28 (5.59, 7.04)	<0.001
Gender				
Female	Ref	Ref	Ref	Ref
Male	0.13 (0.11, 0.16)	<0.001	0.13 (0.11, 0.16)	<0.001
Race				
Mexican American	Ref	Ref	Ref	Ref
Other Hispanic	1.23 (0.86, 1.77)	0.262	1.35 (1.00, 1.83)	0.049
Non-Hispanic White	1.72 (1.36, 2.18)	<0.001	1.70 (1.36, 2.12)	<0.001
Non-Hispanic Black	0.85 (0.64, 1.13)	0.256	0.81 (0.62, 1.06)	0.122
Other Race	1.65 (1.15, 2.37)	0.007	1.53 (1.11, 2.11)	0.010
Smoking				
No	Ref	Ref	Ref	Ref
Yes	1.28 (1.12, 1.46)	<0.001	1.28 (1.14, 1.44)	<0.001

Model 1: multivariable analysis for the association between smoking and osteoporosis, adjusted for age, gender, and race; OR: odds ratio; CI: confidence interval; Ref: reference.

Table S5 Associations of smoking and other variables with osteoporosis based on model 2 among US adults, who responded to smoking status and osteoporosis questionnaires in the National Health and Nutrition Examination Survey (NHANES), data from 1999-2010, 2013–2014, and 2017–2018 (N=30856 for primary analysis; N=37214 for sensitivity analysis)

**Variable**	**Primary results**		**Sensitivity results**	
	**OR (95% CI)**	***P*-value**	**OR (95% CI)**	***P*-value**
Age				
<65	Ref	Ref	Ref	Ref
≥65	5.35 (4.63, 6.18)	<0.001	5.35 (4.71, 6.07)	<0.001
Gender				
Female	Ref	Ref	Ref	Ref
Male	0.14 (0.12, 0.17)	<0.001	0.15 (0.12, 0.17)	<0.001
Race				
Mexican American	Ref	Ref	Ref	Ref
Other Hispanic	1.28 (0.89, 1.84)	0.188	1.39 (1.03, 1.88)	0.034
Non-Hispanic White	1.85 (1.44, 2.40)	<0.001	1.82 (1.43, 2.31)	<0.001
Non-Hispanic Black	0.90 (0.67, 1.21)	0.484	0.85 (0.65, 1.11)	0.220
Other Race	1.78 (1.24, 2.56)	0.002	1.61 (1.16, 2.24)	0.005
Education				
Under high school	Ref	Ref	Ref	Ref
High school or equivalent	1.05 (0.86, 1.28)	0.631	1.05 (0.88, 1.26)	0.588
College or above	0.93 (0.78, 1.11)	0.420	1.01 (0.85, 1.19)	0.925
Not recorded			1.10 (0.35, 3.51)	0.867
Marital status				
Married/Living with partner	Ref	Ref	Ref	Ref
Widowed/divorced/separated	1.26 (1.10, 1.43)	<0.001	1.28 (1.13, 1.45)	<0.001
Never married	0.49 (0.38, 0.64)	<0.001	0.52 (0.42, 0.66)	<0.001
Not recorded			0.50 (0.22, 1.13)	0.096
PIR				
≤1.30	Ref	Ref	Ref	Ref
>1.30 to ≤3.50	0.93 (0.78, 1.11)	0.403	0.89 (0.75, 1.04)	0.144
>3.50	0.84 (0.69, 1.03)	0.098	0.76 (0.63, 0.92)	0.005
Not recorded			0.85 (0.67, 1.08)	0.181
Smoking				
No	Ref	Ref	Ref	Ref
Yes	1.22 (1.07, 1.39)	0.003	1.23 (1.09, 1.38)	<0.001

Model 2: multivariable analysis for the association between smoking and osteoporosis, adjusted for age, gender, race, education, marital status, and ratio of family income to poverty (PIR). PIR: ratio

of family income to poverty; OR: odds ratio; CI: confidence interval; Ref: reference.

Table S6 Associations of smoking and other variables with osteoporosis based on model 3 among US adults, who responded to smoking status and osteoporosis questionnaires in the National Health and Nutrition Examination Survey (NHANES), data from 1999-2010, 2013–2014, and 2017–2018 (N=30856 for primary analysis; N=37214 for sensitivity analysis)

**Variable**	**Primary results**		**Sensitivity results**	
	**OR (95% CI)**	***P*-value**	**OR (95% CI)**	***P*-value**
Age				
<65	Ref	Ref	Ref	Ref
≥65	5.38 (4.65, 6.22)	<0.001	5.36 (4.72, 6.09)	<0.001
Gender				
Female	Ref	Ref	Ref	Ref
Male	0.15 (0.12, 0.17)	<0.001	0.15 (0.13, 0.17)	<0.001
Race				
Mexican American	Ref	Ref	Ref	Ref
Other Hispanic	1.26 (0.88, 1.82)	0.210	1.37 (1.01, 1.85)	0.040
Non-Hispanic White	1.81 (1.40, 2.34)	<0.001	1.77 (1.39, 2.25)	<0.001
Non-Hispanic Black	0.90 (0.67, 1.21)	0.497	0.84 (0.64, 1.11)	0.220
Other Race	1.69 (1.16, 2.45)	0.007	1.52 (1.09, 2.12)	0.015
Education				
Under high school	Ref	Ref	Ref	Ref
High school or equivalent	1.05 (0.86, 1.27)	0.627	1.05 (0.88, 1.26)	0.562
College or above	0.93 (0.78, 1.11)	0.397	1.01 (0.85, 1.19)	0.943
Not recorded			1.02 (0.32, 3.30)	0.969
Marital status				
Married/Living with partner	Ref	Ref	Ref	Ref
Widowed/divorced/separated	1.25 (1.10, 1.42)	<0.001	1.28 (1.13, 1.45)	<0.001
Never married	0.48 (0.37, 0.63)	<0.001	0.51 (0.41, 0.65)	<0.001
Not recorded			0.50 (0.22, 1.13)	0.095
PIR				
≤1.30	Ref	Ref	Ref	Ref
>1.30 to ≤3.50	0.93 (0.78, 1.11)	0.403	0.89 (0.76, 1.05)	0.154
>3.50	0.84 (0.69, 1.03)	0.085	0.76 (0.63, 0.92)	0.004
Not recorded			0.85 (0.67, 1.08)	0.175
BMI				
<25	Ref	Ref	Ref	Ref
≥25 to <30	0.84 (0.72, 0.97)	0.019	0.81 (0.71, 0.92)	0.002
≥30	0.81 (0.69, 0.95)	0.010	0.79 (0.69, 0.92)	0.002
Not recorded			1.11 (0.83, 1.50)	0.475

Variable	Primary results		Sensitivity results	
	**OR (95% CI)**	***P*-value**	**OR (95% CI)**	***P*-value**
Calcium				
<9.5	Ref	Ref	Ref	Ref
≥9.5	1.06 (0.94, 1.20)	0.345	1.07 (0.95, 1.20)	0.268
Not recorded			1.03 (0.81, 1.31)	0.814
Smoking				
No	Ref	Ref	Ref	Ref
Yes	1.21 (1.06, 1.39)	0.004	1.22 (1.08, 1.37)	0.001

Model 3: multivariable analysis for the association between smoking and osteoporosis, adjusted for age, gender, race, education, marital status, ratio of family income to poverty (PIR), body mass index (BMI), and calcium. PIR: ratio of family income to poverty; BMI: body mass index; OR: odds ratio; CI: confidence interval; Ref: reference.

Table S7 Baseline characteristics of participants with hip osteoporotic fracture for primary analysis among US adults, who responded to smoking status and hip osteoporotic fracture questionnaires in the National Health and Nutrition Examination Survey (NHANES), data from 1999-2010, 2013–2014, and 2017–2018 (N=30928)

**Variable**	**Total (N=30928)**	**Hip fracture-No (N=30468)**	**Hip fracture-Yes (N=460)**	***P*-value**
Age, N (%)				< 0.001
<65	22540 (72.9)	22327 (80.8)	213 (54.1)	
≥65	8388 (27.1)	8141 (19.2)	247 (45.9)	
Gender, N (%)				0.838
Female	15971 (51.6)	15738 (51.9)	233 (52.5)	
Male	14957 (48.4)	14730 (48.1)	227 (47.5)	
Race, N (%)				0.182
Mexican American	5758 (18.6)	5688 (7.4)	70 (6.0)	
Other Hispanic	2102 (6.8)	2083 (4.8)	19 (3.0)	
Non-Hispanic White	15279 (49.4)	15003 (71.8)	276 (77.3)	
Non-Hispanic Black	5930 (19.2)	5857 (10.2)	73 (9.5)	
Other Race	1859 (6.0)	1837 (5.8)	22 (4.2)	
Education, N (%)				< 0.001
Under high school	8709 (28.2)	8538 (17.8)	171 (27.7)	
High school or equivalent	7303 (23.6)	7192 (24.9)	111 (26.0)	
College or above	14916 (48.2)	14738 (57.2)	178 (46.3)	
Marital status, N (%)				< 0.001
Married/Living with partner	19185 (62)	18952 (65.6)	233 (54.3)	
Widowed/divorced/separated	7288 (23.6)	7103 (19.7)	185 (36.0)	
Never married	4455 (14.4)	4413 (14.7)	42 (9.7)	
PIR, N (%)				< 0.001
≤1.30	9093 (29.4)	8903 (19.9)	190 (30.7)	
>1.30 to ≤3.50	11888 (38.4)	11728 (36.0)	160 (34.0)	
>3.50	9947 (32.2)	9837 (44.0)	110 (35.3)	
BMI, N (%)				0.099
<25	9073 (29.3)	8909 (31.3)	164 (34.5)	
≥25 to <30	10804 (34.9)	10633 (34.0)	171 (37.1)	
≥30	11051 (35.7)	10926 (34.6)	125 (28.5)	
Calcium, N (%)				0.857
<9.5	16264 (52.6)	16027 (50.5)	237 (51.1)	
≥9.5	14664 (47.4)	14441 (49.5)	223 (48.9)	
Smoking, N (%)				< 0.001

Variable	Total (N=30928)	Hip fracture-No (N=30468)	Hip fracture-Yes (N=460)	*P*-value
No	16138 (52.2)	15947 (52.2)	191 (39.9)	
Yes	14790 (47.8)	14521 (47.8)	269 (60.1)	

PIR: ratio of family income to poverty; BMI: body mass index.

Table S8 Baseline characteristics of participants with hip osteoporotic fracture for sensitivity analysis among US adults, who responded to smoking status and hip osteoporotic fracture questionnaires in the National Health and Nutrition Examination Survey (NHANES), data from 1999-2010, 2013–2014, and 2017–2018 (N=37298)

**Variable**	**Total (N=37298)**	**Hip fracture-No (N=36719)**	**Hip fracture-Yes (N=579)**	***P*-value**
Age, N (%)				< 0.001
<65	26806 (71.9)	26554 (80.2)	252 (50.3)	
≥65	10492 (28.1)	10165 (19.8)	327 (49.7)	
Gender, N (%)				0.615
Female	19395 (52.0)	19096 (52.2)	299 (53.6)	
Male	17903 (48.0)	17623 (47.8)	280 (46.4)	
Race, N (%)				0.501
Mexican American	7049 (18.9)	6962 (7.6)	87 (6.0)	
Other Hispanic	2651 (7.1)	2620 (5.1)	31 (4.5)	
Non-Hispanic White	17770 (47.6)	17434 (70.4)	336 (74.2)	
Non-Hispanic Black	7564 (20.3)	7468 (11.0)	96 (10.3)	
Other Race	2264 (6.1)	2235 (5.9)	29 (5.0)	
Education, N (%)				< 0.001
Under high school	10989 (29.5)	10767 (18.7)	222 (30.0)	
High school or equivalent	8856 (23.7)	8720 (25.2)	136 (24.7)	
College or above	17392 (46.6)	17174 (56.0)	218 (45.0)	
Not recorded	61 (0.2)	58 (0.1)	3 (0.3)	
Marital status, N (%)				< 0.001
Married/Living with partner	22433 (60.1)	22160 (63.7)	273 (50.7)	
Widowed/divorced/separated	8881 (23.8)	8639 (19.6)	242 (37.8)	
Never married	5489 (14.7)	5432 (15.2)	57 (10.2)	
Not recorded	495 (1.3)	488 (1.5)	7 (1.3)	
PIR, N (%)				< 0.001
≤1.30	10103 (27.1)	9887 (18.8)	216 (28.9)	
>1.30 to ≤3.50	13166 (35.3)	12971 (33.7)	195 (33.7)	
>3.50	10736 (28.8)	10608 (40.1)	128 (32.0)	
Not recorded	3293 (8.8)	3253 (7.4)	40 (5.4)	
BMI, N (%)				< 0.001
<25	10774 (28.9)	10579 (31.1)	195 (32.5)	
≥25 to <30	12689 (34.0)	12501 (33.3)	188 (33.1)	
≥30	12964 (34.8)	12818 (33.8)	146 (26.5)	
Not recorded	871 (2.3)	821 (1.8)	50 (7.9)	

Variable	Total (N=37298)	Hip fracture-No (N=36719)	Hip fracture-Yes (N=579)	*P*-value
Calcium, N (%)				0.369
<9.5	18603 (49.9)	18315 (48.4)	288 (49.8)	
≥9.5	16308 (43.7)	16061 (46.1)	247 (43.6)	
Not recorded	2387 (6.4)	2343 (5.5)	44 (6.7)	
Smoking, N (%)				< 0.001
No	19599 (52.5)	19351 (52.3)	248 (41.7)	
Yes	17699 (47.5)	17368 (47.7)	331 (58.3)	

PIR: ratio of family income to poverty; BMI: body mass index.

Table S9 Associations of all the included variables with hip osteoporotic fracture based on model 0 among US adults, who responded to smoking status and hip osteoporotic fracture questionnaires in the National Health and Nutrition Examination Survey (NHANES), data from 1999-2010, 2013–2014, and 2017–2018 (N=30928 for primary analysis; N=37298 for sensitivity analysis)

**Variable**	**Primary results**		**Sensitivity results**	
	**OR (95% CI)**	***P*-value**	**OR (95% CI)**	***P*-value**
Age				
<65	Ref	Ref	Ref	Ref
≥65	3.57 (2.87, 4.45)	<0.001	4.01 (3.26, 4.92)	<0.001
Gender				
Female	Ref	Ref	Ref	Ref
Male	0.98 (0.78, 1.23)	0.838	0.95 (0.76, 1.18)	0.615
Race				
Mexican American	Ref	Ref	Ref	Ref
Other Hispanic	0.76 (0.32, 1.83)	0.542	1.14 (0.56, 2.31)	0.725
Non-Hispanic White	1.33 (0.92, 1.91)	0.125	1.34 (0.93, 1.92)	0.110
Non-Hispanic Black	1.14 (0.78, 1.69)	0.492	1.18 (0.82, 1.69)	0.362
Other Race	0.89 (0.48, 1.65)	0.712	1.07 (0.65, 1.78)	0.785
Education				
Under high school	Ref	Ref	Ref	Ref
High school or equivalent	0.67 (0.47, 0.95)	0.024	0.61 (0.46, 0.81)	<0.001
College or above	0.52 (0.39, 0.69)	<0.001	0.50 (0.39, 0.64)	<0.001
Not recorded			1.54 (0.36, 6.66)	0.560
Marital status				
Married/Living with partner	Ref	Ref	Ref	Ref
Widowed/divorced/separated	2.21 (1.71, 2.87)	<0.001	2.42 (1.91, 3.05)	<0.001
Never married	0.80 (0.54, 1.18)	0.258	0.85 (0.61, 1.19)	0.338
Not recorded			1.05 (0.43, 2.59)	0.915
PIR				
≤1.30	Ref	Ref	Ref	Ref
>1.30 to ≤3.50	0.61 (0.46, 0.81)	<0.001	0.65 (0.50, 0.85)	0.002
>3.50	0.52 (0.37, 0.72)	<0.001	0.52 (0.38, 0.71)	<0.001
Not recorded			0.48 (0.32, 0.70)	<0.001
BMI				
<25	Ref	Ref	Ref	Ref
≥25 to <30	0.99 (0.75, 1.30)	0.935	0.95 (0.73, 1.24)	0.716
≥30	0.75 (0.55, 1.01)	0.054	0.75 (0.57, 0.99)	0.042
Not recorded			4.14 (2.68, 6.40)	<0.001

Variable	Primary results		Sensitivity results	
	**OR (95% CI)**	***P*-value**	**OR (95% CI)**	***P*-value**
Calcium				
<9.5	Ref	Ref	Ref	Ref
≥9.5	0.98 (0.78, 1.23)	0.857	0.92 (0.74, 1.14)	0.431
Not recorded			1.18 (0.84, 1.66)	0.345
Smoking				
No	Ref	Ref	Ref	Ref
Yes	1.65 (1.28, 2.11)	<0.001	1.53 (1.22, 1.92)	<0.001

Model 0: univariate analysis; PIR: ratio of family income to poverty; BMI: body mass index; OR: odds ratio; CI: confidence interval; Ref: reference.

Table S10 Associations of smoking and other variables with hip osteoporotic fracture based on model 1 among US adults, who responded to smoking status and hip osteoporotic fracture questionnaires in the National Health and Nutrition Examination Survey (NHANES), data from 1999-2010, 2013–2014, and 2017–2018 (N=30928 for primary analysis; N=37298 for sensitivity analysis)

**Variable**	**Primary results**		**Sensitivity results**	
	**OR (95% CI)**	***P*-value**	**OR (95% CI)**	***P*-value**
Age				
<65	Ref	Ref	Ref	Ref
≥65	3.48 (2.78, 4.37)	<0.001	3.97 (3.21, 4.90)	<0.001
Gender				
Female	Ref	Ref	Ref	Ref
Male	0.94 (0.74, 1.20)	0.614	0.93 (0.74, 1.18)	0.561
Race				
Mexican American	Ref	Ref	Ref	Ref
Other Hispanic	0.71 (0.29, 1.70)	0.435	1.03 (0.51, 2.07)	0.928
Non-Hispanic White	0.99 (0.69, 1.43)	0.964	0.97 (0.68, 1.39)	0.872
Non-Hispanic Black	1.01 (0.68, 1.50)	0.960	1.02 (0.71, 1.46)	0.912
Other Race	0.83 (0.45, 1.54)	0.546	0.99 (0.59, 1.64)	0.957
Smoking				
No	Ref	Ref	Ref	Ref
Yes	1.60 (1.24, 2.08)	<0.001	1.51 (1.19, 1.91)	<0.001

Model 1: multivariable analysis for the association between smoking and osteoporosis, adjusted for age, gender, and race; OR: odds ratio; CI: confidence interval; Ref: reference.

Table S11 Associations of smoking and other variables with hip osteoporotic fracture based on model 2 among US adults, who responded to smoking status and hip osteoporotic fracture questionnaires in the National Health and Nutrition Examination Survey (NHANES), data from 1999-2010, 2013–2014, and 2017–2018 (N=30928 for primary analysis; N=37298 for sensitivity analysis)

**Variable**	**Primary results**		**Sensitivity results**	
	**OR (95% CI)**	***P*-value**	**OR (95% CI)**	***P*-value**
Age				
<65	Ref	Ref	Ref	Ref
≥65	3.04 (2.36, 3.91)	<0.001	3.43 (2.70, 4.35)	<0.001
Gender				
Female	Ref	Ref	Ref	Ref
Male	1.07 (0.83, 1.39)	0.600	1.07 (0.84, 1.37)	0.576
Race				
Mexican American	Ref	Ref	Ref	Ref
Other Hispanic	0.74 (0.31, 1.78)	0.503	1.08 (0.55, 2.12)	0.813
Non-Hispanic White	1.30 (0.89, 1.90)	0.180	1.26 (0.86, 1.83)	0.230
Non-Hispanic Black	1.09 (0.73, 1.60)	0.679	1.08 (0.75, 1.54)	0.688
Other Race	1.00 (0.54, 1.88)	0.992	1.20 (0.71, 2.02)	0.488
Education				
Under high school	Ref	Ref	Ref	Ref
High school or equivalent	0.77 (0.54, 1.12)	0.173	0.71 (0.52, 0.96)	0.027
College or above	0.72 (0.51, 1.01)	0.059	0.68 (0.51, 0.91)	0.010
Not recorded			1.31 (0.28, 6.07)	0.731
Marital status				
Married/Living with partner	Ref	Ref	Ref	Ref
Widowed/divorced/separated	1.58 (1.17, 2.13)	0.003	1.69 (1.31, 2.19)	<0.001
Never married	0.99 (0.65, 1.49)	0.953	1.10 (0.77, 1.57)	0.602
Not recorded			1.11 (0.45, 2.75)	0.813
PIR				
≤1.30	Ref	Ref	Ref	Ref
>1.30 to ≤3.50	0.60 (0.44, 0.81)	<0.001	0.67 (0.50, 0.88)	0.004
>3.50	0.66 (0.45, 0.98)	0.039	0.72 (0.51, 1.02)	0.062
Not recorded			0.50 (0.34, 0.74)	<0.001
Smoking				
No	Ref	Ref	Ref	Ref
Yes	1.47 (1.14, 1.91)	0.004	1.39 (1.09, 1.77)	0.007

Model 2: multivariable analysis for the association between smoking and osteoporosis, adjusted for age, gender, race, education, marital status, and ratio of family income to poverty (PIR). PIR: ratio of family income to poverty; OR: odds ratio; CI: confidence interval; Ref: reference.

Table S12 Associations of smoking and other variables with hip osteoporotic fracture based on model 3 among US adults, who responded to smoking status and hip osteoporotic fracture questionnaires in the National Health and Nutrition Examination Survey (NHANES), data from 1999-2010, 2013–2014, and 2017–2018 (N=30928 for primary analysis; N=37298 for sensitivity analysis)

Variable	Primary results		Sensitivity results	
	OR (95% CI)	*P*-value	OR (95% CI)	*P*-value
Age				
<65	Ref	Ref	Ref	Ref
≥65	3.03 (2.35, 3.90)	<0.001	3.28 (2.61, 4.13)	<0.001
Gender				
Female	Ref	Ref	Ref	Ref
Male	1.07 (0.83, 1.39)	0.602	1.07 (0.84, 1.37)	0.554
Race				
Mexican American	Ref	Ref	Ref	Ref
Other Hispanic	0.74 (0.31, 1.76)	0.490	1.03 (0.54, 1.95)	0.929
Non-Hispanic White	1.29 (0.87, 1.90)	0.204	1.23 (0.84, 1.82)	0.287
Non-Hispanic Black	1.11 (0.75, 1.65)	0.594	1.08 (0.74, 1.56)	0.693
Other Race	0.96 (0.51, 1.81)	0.894	1.14 (0.66, 1.95)	0.636
Education				
Under high school	Ref	Ref	Ref	Ref
High school or equivalent	0.78 (0.54, 1.13)	0.182	0.72 (0.53, 0.98)	0.038
College or above	0.72 (0.51, 1.01)	0.058	0.69 (0.52, 0.92)	0.013
Not recorded			1.06 (0.20, 5.55)	0.945
Marital status				
Married/Living with partner	Ref	Ref	Ref	Ref
Widowed/divorced/separated	1.58 (1.17, 2.13)	0.003	1.65 (1.27, 2.14)	<0.001
Never married	0.97 (0.64, 1.46)	0.878	1.05 (0.74, 1.49)	0.782
Not recorded			1.07 (0.43, 2.68)	0.885
PIR				
≤1.30	Ref	Ref	Ref	Ref
>1.30 to ≤3.50	0.60 (0.45, 0.81)	<0.001	0.68 (0.52, 0.90)	0.006
>3.50	0.66 (0.45, 0.98)	0.038	0.74 (0.52, 1.05)	0.087
Not recorded			0.50 (0.34, 0.74)	<0.001
BMI				
<25	Ref	Ref	Ref	Ref
≥25 to <30	0.94 (0.71, 1.23)	0.633	0.89 (0.69, 1.16)	0.404
≥30	0.72 (0.53, 0.99)	0.045	0.73 (0.55, 0.97)	0.031

Variable	Primary results		Sensitivity results	
	OR (95% CI)	*P*-value	OR (95% CI)	*P*-value
Not recorded			2.64 (1.76, 3.95)	<0.001
Calcium				
<9.5	Ref	Ref	Ref	Ref
≥9.5	0.94 (0.74, 1.20)	0.631	0.90 (0.72, 1.13)	0.377
Not recorded			1.02 (0.72, 1.44)	0.914
Smoking				
No	Ref	Ref	Ref	Ref
Yes	1.47 (1.14, 1.90)	0.004	1.39 (1.10, 1.76)	0.007

Model 3: multivariable analysis for the association between smoking and osteoporosis, adjusted for age, gender, race, education, marital status, ratio of family income to poverty (PIR), body mass index (BMI), and calcium. PIR: ratio of family income to poverty; BMI: body mass index; OR: odds ratio; CI: confidence interval; Ref: reference.

Table S13 Baseline characteristics of participants with wrist osteoporotic fracture for primary analysis among US adults, who responded to smoking status and wrist osteoporotic fracture questionnaires in the National Health and Nutrition Examination Survey (NHANES), data from 1999-2010, 2013–2014, and 2017–2018 (N=30923)

**Variable**	**Total (N=30923)**	**Wrist fracture-No (N=28076)**	**Wrist fracture-Yes (N=2847)**	***P*-value**
Age, N (%)				0.003
<65	22533 (72.9)	20554 (80.7)	1979 (78.3)	
≥65	8390 (27.1)	7522 (19.3)	868 (21.7)	
Gender, N (%)				< 0.001
Female	15972 (51.7)	14660 (52.8)	1312 (44.6)	
Male	14951 (48.3)	13416 (47.2)	1535 (55.4)	
Race, N (%)				< 0.001
Mexican American	5760 (18.6)	5424 (7.8)	336 (4.1)	
Other Hispanic	2099 (6.8)	1954 (5.0)	145 (2.8)	
Non-Hispanic White	15277 (49.4)	13361 (70.4)	1916 (84.0)	
Non-Hispanic Black	5928 (19.2)	5595 (10.8)	333 (5.3)	
Other Race	1859 (6.0)	1742 (6.0)	117 (3.9)	
Education, N (%)				0.005
Under high school	8710 (28.2)	8042 (18.2)	668 (16.0)	
High school or equivalent	7299 (23.6)	6626 (25.1)	673 (23.8)	
College or above	14914 (48.2)	13408 (56.7)	1506 (60.2)	
Marital status, N (%)				0.401
Married/Living with partner	19181 (62)	17478 (65.6)	1703 (64.6)	
Widowed/divorced/separated	7289 (23.6)	6553 (19.8)	736 (21.0)	
Never married	4453 (14.4)	4045 (14.7)	408 (14.5)	
PIR, N (%)				0.390
≤1.30	9096 (29.4)	8248 (20.1)	848 (19.4)	
>1.30 to ≤3.50	11881 (38.4)	10849 (36.1)	1032 (35.2)	
>3.50	9946 (32.2)	8979 (43.8)	967 (45.4)	
BMI, N (%)				0.964
<25	9063 (29.3)	8204 (31.3)	859 (31.3)	
≥25 to <30	10810 (35.0)	9839 (34.1)	971 (33.8)	
≥30	11050 (35.7)	10033 (34.6)	1017 (34.8)	
Calcium, N (%)				0.702
<9.5	16261 (52.6)	14794 (50.6)	1467 (50.2)	
≥9.5	14662 (47.4)	13282 (49.4)	1380 (49.8)	
Smoking, N (%)				< 0.001

Variable	Total (N=30923)	Wrist fracture-No (N=28076)	Wrist fracture-Yes (N=2847)	*P*-value
No	16141 (52.2)	14908 (53.0)	1233 (43.9)	
Yes	14782 (47.8)	13168 (47.0)	1614 (56.1)	

PIR: ratio of family income to poverty; BMI: body mass index.

Table S14 Baseline characteristics of participants with wrist osteoporotic fracture for sensitivity analysis among US adults, who responded to smoking status and wrist osteoporotic fracture questionnaires in the National Health and Nutrition Examination Survey (NHANES), data from 1999-2010, 2013–2014, and 2017–2018 (N=37297)

**Variable**	**Total (N=37297)**	**Wrist fracture-No (N=33930)**	**Wrist fracture-Yes (N=3367)**	***P*-value**
Age, N (%)				0.001
<65	26802 (71.9)	24486 (80.1)	2316 (77.6)	
≥65	10495 (28.1)	9444 (19.9)	1051 (22.4)	
Gender, N (%)				< 0.001
Female	19400 (52.0)	17836 (53.1)	1564 (44.8)	
Male	17897 (48.0)	16094 (46.9)	1803 (55.2)	
Race, N (%)				< 0.001
Mexican American	7055 (18.9)	6635 (8.0)	420 (4.2)	
Other Hispanic	2648 (7.1)	2468 (5.3)	180 (3.1)	
Non-Hispanic White	17768 (47.6)	15574 (69.0)	2194 (82.8)	
Non-Hispanic Black	7562 (20.3)	7128 (11.6)	434 (5.9)	
Other Race	2264 (6.1)	2125 (6.2)	139 (3.9)	
Education, N (%)				0.002
Under high school	10991 (29.5)	10160 (19.2)	831 (16.6)	
High school or equivalent	8853 (23.7)	8047 (25.2)	806 (24.5)	
College or above	17391 (46.6)	15665 (55.5)	1726 (58.9)	
Not recorded	62 (0.2)	58 (0.1)	4 (0.1)	
Marital status, N (%)				0.292
Married/Living with partner	22429 (60.1)	20482 (63.6)	1947 (62.3)	
Widowed/divorced/separated	8882 (23.8)	7994 (19.7)	888 (21.4)	
Never married	5491 (14.7)	5004 (15.1)	487 (14.8)	
Not recorded	495 (1.3)	450 (1.5)	45 (1.5)	
PIR, N (%)				0.181
≤1.30	10109 (27.1)	9177 (19.0)	932 (18.6)	
>1.30 to ≤3.50	13160 (35.3)	12015 (33.7)	1145 (33.6)	
>3.50	10736 (28.8)	9715 (39.9)	1021 (41.4)	
Not recorded	3292 (8.8)	3023 (7.5)	269 (6.3)	
BMI, N (%)				0.947
<25	10766 (28.9)	9762 (31.0)	1004 (31.5)	
≥25 to <30	12692 (34.0)	11574 (33.4)	1118 (33.0)	
≥30	12965 (34.8)	11809 (33.7)	1156 (33.6)	
Not recorded	874 (2.3)	785 (1.9)	89 (1.9)	

**Variable**	**Total (N=37297)**	**Wrist fracture-No (N=33930)**	**Wrist fracture-Yes (N=3367)**	***P*-value**
Calcium, N (%)				0.037
<9.5	18601 (49.9)	16930 (48.3)	1671 (48.9)	
≥9.5	16307 (43.7)	14790 (46.0)	1517 (46.7)	
Not recorded	2389 (6.4)	2210 (5.7)	179 (4.4)	
Smoking, N (%)				< 0.001
No	19603 (52.6)	18157 (53.2)	1446 (43.1)	
Yes	17694 (47.4)	15773 (46.8)	1921 (56.9)	

PIR: ratio of family income to poverty; BMI: body mass index.

Table S15 Associations of all the included variables with wrist osteoporotic fracture based on model 0 among US adults, who responded to smoking status and wrist osteoporotic fracture questionnaires in the National Health and Nutrition Examination Survey (NHANES), data from 1999-2010, 2013–2014, and 2017–2018 (N=30923 for primary analysis; N=37297 for sensitivity analysis)

**Variable**	**Primary results**		**Sensitivity results**	
	**OR (95% CI)**	***P*-value**	**OR (95% CI)**	***P*-value**
Age				
<65	Ref	Ref	Ref	Ref
≥65	1.17 (1.05, 1.29)	0.003	1.16 (1.06, 1.26)	0.001
Gender				
Female	Ref	Ref	Ref	Ref
Male	1.39 (1.26, 1.52)	<0.001	1.39 (1.27, 1.52)	<0.001
Race				
Mexican American	Ref	Ref	Ref	Ref
Other Hispanic	1.07 (0.85, 1.36)	0.548	1.10 (0.89, 1.36)	0.374
Non-Hispanic White	2.30 (1.97, 2.68)	<0.001	2.25 (1.96, 2.59)	<0.001
Non-Hispanic Black	0.94 (0.78, 1.12)	0.479	0.96 (0.82, 1.12)	0.582
Other Race	1.27 (0.96, 1.67)	0.097	1.20 (0.93, 1.54)	0.169
Education				
Under high school	Ref	Ref	Ref	Ref
High school or equivalent	1.08 (0.94, 1.24)	0.253	1.12 (0.98, 1.28)	0.086
College or above	1.21 (1.06, 1.38)	0.005	1.23 (1.09, 1.38)	0.001
Not recorded			0.54 (0.17, 1.71)	0.291
Marital status				
Married/Living with partner	Ref	Ref	Ref	Ref
Widowed/divorced/separated	1.08 (0.97, 1.20)	0.180	1.11 (1.01, 1.22)	0.035
Never married	1.00 (0.88, 1.14)	0.948	1.00 (0.89, 1.12)	0.972
Not recorded			1.02 (0.68, 1.55)	0.916
PIR				
≤1.30	Ref	Ref	Ref	Ref
>1.30 to ≤3.50	1.01 (0.90, 1.13)	0.873	1.02 (0.91, 1.14)	0.709
>3.50	1.07 (0.95, 1.21)	0.274	1.06 (0.95, 1.19)	0.306
Not recorded			0.86 (0.71, 1.06)	0.155
BMI				
<25	Ref	Ref	Ref	Ref
≥25 to <30	0.99 (0.87, 1.14)	0.902	0.97 (0.86, 1.10)	0.664
≥30	1.01 (0.89, 1.14)	0.895	0.98 (0.87, 1.10)	0.737

Variable	Primary results		Sensitivity results	
	OR (95% CI)	*P*-value	OR (95% CI)	*P*-value
Not recorded			0.98 (0.78, 1.23)	0.856
Calcium				
<9.5	Ref	Ref	Ref	Ref
≥9.5	1.02 (0.93, 1.12)	0.702	1.00 (0.91, 1.10)	0.961
Not recorded			0.77 (0.63, 0.93)	0.008
Smoking				
No	Ref	Ref	Ref	Ref
Yes	1.44 (1.30, 1.61)	<0.001	1.51 (1.36, 1.67)	<0.001

Model 0: univariate analysis; PIR: ratio of family income to poverty; BMI: body mass index; OR: odds ratio; CI: confidence interval; Ref: reference.

Table S16 Associations of smoking and other variables with wrist osteoporotic fracture based on model 1 among US adults, who responded to smoking status and wrist osteoporotic fracture questionnaires in the National Health and Nutrition Examination Survey (NHANES), data from 1999-2010, 2013–2014, and 2017–2018 (N=30923 for primary analysis; N=37297 for sensitivity analysis)

**Variable**	**Primary results**		**Sensitivity results**	
	**OR (95% CI)**	***P*-value**	**OR (95% CI)**	***P*-value**
Age				
<65	Ref	Ref	Ref	Ref
≥65	1.08 (0.97, 1.19)	0.148	1.07 (0.98, 1.17)	0.105
Gender				
Female	Ref	Ref	Ref	Ref
Male	1.34 (1.21, 1.48)	<0.001	1.34 (1.22, 1.47)	<0.001
Race				
Mexican American	Ref	Ref	Ref	Ref
Other Hispanic	1.09 (0.86, 1.37)	0.486	1.12 (0.91, 1.38)	0.289
Non-Hispanic White	2.24 (1.92, 2.63)	<0.001	2.19 (1.89, 2.53)	<0.001
Non-Hispanic Black	0.95 (0.79, 1.14)	0.581	0.97 (0.83, 1.14)	0.690
Other Race	1.28 (0.97, 1.69)	0.077	1.22 (0.95, 1.57)	0.125
Smoking				
No	Ref	Ref	Ref	Ref
Yes	1.32 (1.18, 1.49)	<0.001	1.37 (1.23, 1.53)	<0.001

Model 1: multivariable analysis for the association between smoking and osteoporosis, adjusted for age, gender, and race; OR: odds ratio; CI: confidence interval; Ref: reference.

Table S17 Associations of smoking and other variables with wrist osteoporotic fracture based on model 2 among US adults, who responded to smoking status and wrist osteoporotic fracture questionnaires in the National Health and Nutrition Examination Survey (NHANES), data from 1999-2010, 2013–2014, and 2017–2018 (N=30923 for primary analysis; N=37297 for sensitivity analysis)

**Variable**	**Primary results**		**Sensitivity results**	
	**OR (95% CI)**	***P*-value**	**OR (95% CI)**	***P*-value**
Age				
<65	Ref	Ref	Ref	Ref
≥65	1.08 (0.96, 1.21)	0.190	1.07 (0.97, 1.18)	0.177
Gender				
Female	Ref	Ref	Ref	Ref
Male	1.37 (1.24, 1.52)	<0.001	1.37 (1.24, 1.51)	<0.001
Race				
Mexican American	Ref	Ref	Ref	Ref
Other Hispanic	1.06 (0.84, 1.35)	0.602	1.09 (0.89, 1.35)	0.388
Non-Hispanic White	2.26 (1.92, 2.67)	<0.001	2.18 (1.86, 2.55)	<0.001
Non-Hispanic Black	0.92 (0.76, 1.12)	0.404	0.93 (0.79, 1.10)	0.407
Other Race	1.27 (0.95, 1.69)	0.103	1.20 (0.92, 1.56)	0.182
Education				
Under high school	Ref	Ref	Ref	Ref
High school or equivalent	0.95 (0.83, 1.10)	0.487	0.98 (0.86, 1.13)	0.785
College or above	1.12 (0.97, 1.30)	0.117	1.13 (0.99, 1.30)	0.072
Not recorded			0.54 (0.16, 1.84)	0.324
Marital status				
Married/Living with partner	Ref	Ref	Ref	Ref
Widowed/divorced/separated	1.13 (1.00, 1.28)	0.059	1.16 (1.04, 1.30)	0.008
Never married	1.11 (0.96, 1.28)	0.152	1.10 (0.97, 1.26)	0.133
Not recorded			1.06 (0.69, 1.63)	0.790
PIR				
≤1.30	Ref	Ref	Ref	Ref
>1.30 to ≤3.50	0.89 (0.79, 1.00)	0.049	0.91 (0.81, 1.01)	0.077
>3.50	0.86 (0.74, 0.99)	0.035	0.86 (0.76, 0.98)	0.026
Not recorded			0.82 (0.66, 1.00)	0.052
Smoking				
No	Ref	Ref	Ref	Ref
Yes	1.33 (1.18, 1.49)	<0.001	1.38 (1.23, 1.54)	<0.001

Model 2: multivariable analysis for the association between smoking and osteoporosis, adjusted for

age, gender, race, education, marital status, and ratio of family income to poverty (PIR). PIR: ratio of family income to poverty; OR: odds ratio; CI: confidence interval; Ref: reference.

Table S18 Associations of smoking and other variables with wrist osteoporotic fracture based on model 3 among US adults, who responded to smoking status and wrist osteoporotic fracture questionnaires in the National Health and Nutrition Examination Survey (NHANES), data from 1999-2010, 2013–2014, and 2017–2018 (N=30923 for primary analysis; N=37297 for sensitivity analysis)

**Variable**	**Primary results**		**Sensitivity results**	
	**OR (95% CI)**	***P*-value**	**OR (95% CI)**	***P*-value**
Age				
<65	Ref	Ref	Ref	Ref
≥65	1.08 (0.97, 1.21)	0.178	1.07 (0.97, 1.18)	0.163
Gender				
Female	Ref	Ref	Ref	Ref
Male	1.38 (1.24, 1.53)	<0.001	1.38 (1.25, 1.52)	<0.001
Race				
Mexican American	Ref	Ref	Ref	Ref
Other Hispanic	1.07 (0.84, 1.36)	0.585	1.10 (0.89, 1.35)	0.372
Non-Hispanic White	2.27 (1.92, 2.68)	<0.001	2.18 (1.86, 2.55)	<0.001
Non-Hispanic Black	0.92 (0.76, 1.12)	0.391	0.94 (0.79, 1.11)	0.468
Other Race	1.27 (0.96, 1.70)	0.098	1.20 (0.92, 1.56)	0.179
Education				
Under high school	Ref	Ref	Ref	Ref
High school or equivalent	0.95 (0.83, 1.10)	0.484	0.98 (0.86, 1.13)	0.786
College or above	1.12 (0.97, 1.30)	0.118	1.13 (0.99, 1.29)	0.076
Not recorded			0.55 (0.16, 1.85)	0.327
Marital status				
Married/Living with partner	Ref	Ref	Ref	Ref
Widowed/divorced/separated	1.13 (1.00, 1.28)	0.059	1.17 (1.04, 1.30)	0.008
Never married	1.11 (0.96, 1.28)	0.144	1.11 (0.98, 1.26)	0.113
Not recorded			1.05 (0.68, 1.62)	0.812
PIR				
≤1.30	Ref	Ref	Ref	Ref
>1.30 to ≤3.50	0.89 (0.79, 1.00)	0.049	0.91 (0.81, 1.01)	0.079
>3.50	0.86 (0.75, 0.99)	0.036	0.86 (0.76, 0.98)	0.026
Not recorded			0.82 (0.67, 1.01)	0.061
BMI				
<25	Ref	Ref	Ref	Ref
≥25 to <30	0.97 (0.85, 1.11)	0.625	0.95 (0.84, 1.07)	0.399
≥30	1.03 (0.91, 1.17)	0.593	1.00 (0.90, 1.13)	0.935

Variable	Primary results		Sensitivity results	
	**OR (95% CI)**	***P*-value**	**OR (95% CI)**	***P*-value**
Not recorded			0.98 (0.77, 1.26)	0.901
Calcium				
<9.5	Ref	Ref	Ref	Ref
≥9.5	0.98 (0.89, 1.08)	0.628	0.96 (0.87, 1.06)	0.384
Not recorded			0.82 (0.67, 1.00)	0.053
Smoking				
No	Ref	Ref	Ref	Ref
Yes	1.33 (1.18, 1.49)	<0.001	1.38 (1.23, 1.54)	<0.001

Model 3: multivariable analysis for the association between smoking and osteoporosis, adjusted for age, gender, race, education, marital status, ratio of family income to poverty (PIR), body mass index (BMI), and calcium. PIR: ratio of family income to poverty; BMI: body mass index; OR: odds ratio; CI: confidence interval; Ref: reference.

Table S19 Baseline characteristics of participants with spine osteoporotic fracture for primary analysis among US adults, who responded to smoking status and spine osteoporotic fracture questionnaires in the National Health and Nutrition Examination Survey (NHANES), data from 1999-2010, 2013–2014, and 2017–2018 (N=30910)

**Variable**	**Total (N=30910)**	**Spine fracture-No (N=30207)**	**Spine fracture-Yes (N=703)**	***P*-value**
Age, N (%)				< 0.001
<65	22527 (72.9)	22067 (80.7)	460 (74.3)	
≥65	8383 (27.1)	8140 (19.3)	243 (25.7)	
Gender, N (%)				< 0.001
Female	15964 (51.6)	15670 (52.2)	294 (42.7)	
Male	14946 (48.4)	14537 (47.8)	409 (57.3)	
Race, N (%)				< 0.001
Mexican American	5759 (18.6)	5661 (7.5)	98 (4.3)	
Other Hispanic	2100 (6.8)	2061 (4.8)	39 (3.1)	
Non-Hispanic White	15264 (49.4)	14797 (71.6)	467 (82.9)	
Non-Hispanic Black	5927 (19.2)	5873 (10.4)	54 (3.3)	
Other Race	1860 (6.0)	1815 (5.7)	45 (6.4)	
Education, N (%)				0.435
Under high school	8703 (28.2)	8524 (18.0)	179 (15.9)	
High school or equivalent	7298 (23.6)	7115 (24.9)	183 (25.8)	
College or above	14909 (48.2)	14568 (57.1)	341 (58.4)	
Marital status, N (%)				< 0.001
Married/Living with partner	19172 (62.0)	18735 (65.5)	437 (65.9)	
Widowed/divorced/separated	7283 (23.6)	7075 (19.7)	208 (25.2)	
Never married	4455 (14.4)	4397 (14.8)	58 (8.9)	
PIR, N (%)				0.491
≤1.30	9091 (29.4)	8847 (20.0)	244 (22.2)	
>1.30 to ≤3.50	11879 (38.4)	11625 (36.0)	254 (35.2)	
>3.50	9940 (32.2)	9735 (44.0)	205 (42.6)	
BMI, N (%)				0.010
<25	9071 (29.3)	8895 (31.5)	176 (25.3)	
≥25 to <30	10798 (34.9)	10553 (34.0)	245 (34.8)	
≥30	11041 (35.7)	10759 (34.4)	282 (39.8)	
Calcium, N (%)				0.997
<9.5	16251 (52.6)	15887 (50.5)	364 (50.5)	
≥9.5	14659 (47.4)	14320 (49.5)	339 (49.5)	
Smoking, N (%)				< 0.001

Variable	Total (N=30910)	Spine fracture-No (N=30207)	Spine fracture-Yes (N=703)	*P*-value
No	16131 (52.2)	15866 (52.3)	265 (40.7)	
Yes	14779 (47.8)	14341 (47.7)	438 (59.3)	

PIR: ratio of family income to poverty; BMI: body mass index.

Table S20 Baseline characteristics of participants with spine osteoporotic fracture for sensitivity analysis among US adults, who responded to smoking status and spine osteoporotic fracture questionnaires in the National Health and Nutrition Examination Survey (NHANES), data from 1999-2010, 2013–2014, and 2017–2018 (N=37280)

**Variable**	**Total (N=37280)**	**Spine fracture-No (N=36424)**	**Spine fracture-Yes (N=856)**	***P*-value**
Age, N (%)				< 0.001
<65	26796 (71.9)	26243 (80.0)	553 (73.6)	
≥65	10484 (28.1)	10181 (20.0)	303 (26.4)	
Gender, N (%)				< 0.001
Female	19388 (52.0)	19031 (52.5)	357 (42.9)	
Male	17892 (48.0)	17393 (47.5)	499 (57.1)	
Race, N (%)				< 0.001
Mexican American	7053 (18.9)	6931 (7.6)	122 (4.6)	
Other Hispanic	2649 (7.1)	2602 (5.1)	47 (3.3)	
Non-Hispanic White	17753 (47.6)	17196 (70.1)	557 (82.1)	
Non-Hispanic Black	7562 (20.3)	7486 (11.2)	76 (3.8)	
Other Race	2263 (6.1)	2209 (5.9)	54 (6.2)	
Education, N (%)				0.298
Under high school	10981 (29.5)	10753 (18.9)	228 (16.6)	
High school or equivalent	8853 (23.7)	8633 (25.1)	220 (26.1)	
College or above	17384 (46.6)	16977 (55.8)	407 (57.3)	
Not recorded	62 (0.2)	61 (0.1)	1 (0.0)	
Marital status, N (%)				< 0.001
Married/Living with partner	22423 (60.1)	21915 (63.5)	508 (63.1)	
Widowed/divorced/separated	8872 (23.8)	8608 (19.7)	264 (26.6)	
Never married	5491 (14.7)	5418 (15.3)	73 (9.2)	
Not recorded	494 (1.3)	483 (1.5)	11 (1.1)	
PIR, N (%)				0.450
≤1.30	10101 (27.1)	9833 (18.9)	268 (21.2)	
>1.30 to ≤3.50	13157 (35.3)	12860 (33.7)	297 (34.5)	
>3.50	10730 (28.8)	10511 (40.1)	219 (37.8)	
Not recorded	3292 (8.8)	3220 (7.4)	72 (6.5)	
BMI, N (%)				< 0.001
<25	10771 (28.9)	10558 (31.3)	213 (24.6)	
≥25 to <30	12681 (34.0)	12401 (33.3)	280 (33.4)	
≥30	12956 (34.8)	12635 (33.6)	321 (38.2)	
Not recorded	872 (2.3)	830 (1.9)	42 (3.9)	

Variable	Total (N=37280)	Spine fracture-No (N=36424)	Spine fracture-Yes (N=856)	*P*-value
Calcium, N (%)				0.510
<9.5	18589 (49.9)	18169 (48.3)	420 (49.1)	
≥9.5	16303 (43.7)	15914 (46.1)	389 (46.4)	
Not recorded	2388 (6.4)	2341 (5.5)	47 (4.5)	
Smoking, N (%)				< 0.001
No	19592 (52.6)	19264 (52.5)	328 (40.3)	
Yes	17688 (47.4)	17160 (47.5)	528 (59.7)	

PIR: ratio of family income to poverty; BMI: body mass index.

Table S21 Associations of all the included variables with spine osteoporotic fracture based on the model 0 among US adults, who responded to smoking status and spine osteoporotic fracture questionnaires in the National Health and Nutrition Examination Survey (NHANES), data from 1999-2010, 2013–2014, and 2017–2018 (N=30910 for primary analysis; N=37280 for sensitivity analysis)

**Variable**	**Primary results**		**Sensitivity results**	
	**OR (95% CI)**	***P*-value**	**OR (95% CI)**	***P*-value**
Age				
<65	Ref	Ref	Ref	Ref
≥65	1.44 (1.19, 1.74)	<0.001	1.43 (1.20, 1.70)	<0.001
Gender				
Female	Ref	Ref	Ref	Ref
Male	1.46 (1.20, 1.79)	<0.001	1.47 (1.20, 1.79)	<0.001
Race				
Mexican American	Ref	Ref	Ref	Ref
Other Hispanic	1.13 (0.68, 1.86)	0.636	1.09 (0.68, 1.74)	0.719
Non-Hispanic White	2.00 (1.56, 2.57)	<0.001	1.96 (1.56, 2.47)	<0.001
Non-Hispanic Black	0.54 (0.38, 0.77)	<0.001	0.57 (0.41, 0.78)	<0.001
Other Race	1.92 (1.13, 3.26)	0.016	1.77 (1.09, 2.88)	0.023
Education				
Under high school	Ref	Ref	Ref	Ref
High school or equivalent	1.17 (0.90, 1.52)	0.230	1.19 (0.92, 1.52)	0.181
College or above	1.16 (0.91, 1.48)	0.232	1.17 (0.92, 1.49)	0.197
Not recorded			0.18 (0.02, 1.34)	0.093
Marital status				
Married/Living with partner	Ref	Ref	Ref	Ref
Widowed/divorced/separated	1.27 (1.01, 1.60)	0.045	1.36 (1.11, 1.66)	0.003
Never married	0.60 (0.44, 0.82)	0.002	0.61 (0.44, 0.83)	0.002
Not recorded			0.73 (0.31, 1.71)	0.463
PIR				
≤1.30	Ref	Ref	Ref	Ref
>1.30 to ≤3.50	0.88 (0.71, 1.09)	0.227	0.91 (0.75, 1.11)	0.372
>3.50	0.87 (0.69, 1.10)	0.236	0.84 (0.67, 1.06)	0.144
Not recorded			0.79 (0.56, 1.12)	0.190
BMI				
<25	Ref	Ref	Ref	Ref
≥25 to <30	1.27 (1.00, 1.62)	0.050	1.28 (1.02, 1.60)	0.034
≥30	1.44 (1.13, 1.84)	0.004	1.45 (1.16, 1.81)	0.001

Variable	Primary results		Sensitivity results	
	OR (95% CI)	*P*-value	OR (95% CI)	*P*-value
Not recorded			2.65 (1.69, 4.14)	<0.001
Calcium				
<9.5	Ref	Ref	Ref	Ref
≥9.5	1.00 (0.82, 1.22)	0.997	0.99 (0.82, 1.20)	0.936
Not recorded			0.80 (0.57, 1.12)	0.192
Smoking				
No	Ref	Ref	Ref	Ref
Yes	1.60 (1.32, 1.94)	<0.001	1.64 (1.37, 1.96)	<0.001

Model 0: univariate analysis; PIR: ratio of family income to poverty; BMI: body mass index; OR: odds ratio; CI: confidence interval; Ref: reference.

Table S22 Associations of smoking and other variables with spine osteoporotic fracture based on model 1 among US adults, who responded to smoking status and spine osteoporotic fracture questionnaires in the National Health and Nutrition Examination Survey (NHANES), data from 1999-2010, 2013–2014, and 2017–2018 (N=30910 for primary analysis; N=37280 for sensitivity analysis)

**Variable**	**Primary results**		**Sensitivity results**	
	**OR (95% CI)**	***P*-value**	**OR (95% CI)**	***P*-value**
Age				
<65	Ref	Ref	Ref	Ref
≥65	1.36 (1.12, 1.64)	0.002	1.35 (1.13, 1.61)	0.001
Gender				
Female	Ref	Ref	Ref	Ref
Male	1.39 (1.14, 1.70)	0.001	1.39 (1.14, 1.70)	0.001
Race				
Mexican American	Ref	Ref	Ref	Ref
Other Hispanic	1.13 (0.69, 1.86)	0.624	1.10 (0.69, 1.76)	0.694
Non-Hispanic White	1.87 (1.45, 2.41)	<0.001	1.83 (1.44, 2.32)	<0.001
Non-Hispanic Black	0.54 (0.38, 0.77)	<0.001	0.57 (0.41, 0.78)	<0.001
Other Race	1.93 (1.14, 3.29)	0.015	1.79 (1.10, 2.93)	0.021
Smoking				
No	Ref	Ref	Ref	Ref
Yes	1.47 (1.21, 1.78)	<0.001	1.50 (1.25, 1.80)	<0.001

Model 1: multivariable analysis for the association between smoking and osteoporosis, adjusted for age, gender, and race; OR: odds ratio; CI: confidence interval; Ref: reference.

Table S23 Associations of smoking and other variables with spine osteoporotic fracture based on model 2 among US adults, who responded to smoking status and spine osteoporotic fracture questionnaires in the National Health and Nutrition Examination Survey (NHANES), data from 1999-2010, 2013–2014, and 2017–2018 (N=30910 for primary analysis; N=37280 for sensitivity analysis)

**Variable**	**Primary results**		**Sensitivity results**	
	**OR (95% CI)**	***P*-value**	**OR (95% CI)**	***P*-value**
Age				
<65	Ref	Ref	Ref	Ref
≥65	1.24 (1.01, 1.53)	0.041	1.21 (1.00, 1.46)	0.052
Gender				
Female	Ref	Ref	Ref	Ref
Male	1.50 (1.22, 1.84)	<0.001	1.52 (1.24, 1.86)	<0.001
Race				
Mexican American	Ref	Ref	Ref	Ref
Other Hispanic	1.11 (0.68, 1.81)	0.665	1.08 (0.68, 1.71)	0.733
Non-Hispanic White	1.91 (1.48, 2.47)	<0.001	1.85 (1.45, 2.37)	<0.001
Non-Hispanic Black	0.55 (0.38, 0.78)	0.001	0.56 (0.41, 0.77)	<0.001
Other Race	1.93 (1.15, 3.24)	0.013	1.78 (1.10, 2.88)	0.020
Education				
Under high school	Ref	Ref	Ref	Ref
High school or equivalent	1.13 (0.86, 1.48)	0.395	1.13 (0.87, 1.46)	0.367
College or above	1.25 (0.95, 1.63)	0.110	1.25 (0.96, 1.62)	0.095
Not recorded			0.19 (0.02, 1.46)	0.109
Marital status				
Married/Living with partner	Ref	Ref	Ref	Ref
Widowed/divorced/separated	1.28 (1.00, 1.65)	0.051	1.39 (1.12, 1.73)	0.003
Never married	0.66 (0.47, 0.92)	0.014	0.67 (0.48, 0.92)	0.014
Not recorded			0.77 (0.32, 1.83)	0.545
PIR				
≤1.30	Ref	Ref	Ref	Ref
>1.30 to ≤3.50	0.75 (0.60, 0.93)	0.011	0.79 (0.64, 0.96)	0.019
>3.50	0.69 (0.54, 0.87)	0.002	0.68 (0.54, 0.85)	0.001
Not recorded			0.74 (0.52, 1.05)	0.089
Smoking				
No	Ref	Ref	Ref	Ref
Yes	1.42 (1.17, 1.72)	<0.001	1.44 (1.20, 1.73)	<0.001

Model 2: multivariable analysis for the association between smoking and osteoporosis, adjusted for

age, gender, race, education, marital status, and ratio of family income to poverty (PIR). PIR: ratio of family income to poverty; OR: odds ratio; CI: confidence interval; Ref: reference.

Table S24 Associations of smoking and other variables with spine osteoporotic fracture based on model 3 among US adults, who responded to smoking status and spine osteoporotic fracture questionnaires in the National Health and Nutrition Examination Survey (NHANES), data from 1999-2010, 2013–2014, and 2017–2018 (N=30910 for primary analysis; N=37280 for sensitivity analysis)

**Variable**	**Primary results**		**Sensitivity results**	
	**OR (95% CI)**	***P*-value**	**OR (95% CI)**	***P*-value**
Age				
<65	Ref	Ref	Ref	Ref
≥65	1.24 (1.01, 1.53)	0.039	1.18 (0.98, 1.43)	0.078
Gender				
Female	Ref	Ref	Ref	Ref
Male	1.47 (1.20, 1.81)	<0.001	1.49 (1.21, 1.84)	<0.001
Race				
Mexican American	Ref	Ref	Ref	Ref
Other Hispanic	1.13 (0.69, 1.84)	0.631	1.08 (0.68, 1.72)	0.731
Non-Hispanic White	1.95 (1.51, 2.52)	<0.001	1.88 (1.48, 2.40)	<0.001
Non-Hispanic Black	0.54 (0.38, 0.77)	<0.001	0.55 (0.40, 0.77)	<0.001
Other Race	2.06 (1.22, 3.47)	0.007	1.89 (1.16, 3.07)	0.011
Education				
Under high school	Ref	Ref	Ref	Ref
High school or equivalent	1.12 (0.85, 1.47)	0.427	1.12 (0.87, 1.45)	0.375
College or above	1.25 (0.95, 1.64)	0.106	1.26 (0.97, 1.62)	0.082
Not recorded			0.18 (0.02, 1.43)	0.105
Marital status				
Married/Living with partner	Ref	Ref	Ref	Ref
Widowed/divorced/separated	1.29 (1.00, 1.65)	0.047	1.39 (1.11, 1.72)	0.004
Never married	0.68 (0.49, 0.95)	0.024	0.69 (0.50, 0.95)	0.022
Not recorded			0.79 (0.33, 1.88)	0.590
PIR				
≤1.30	Ref	Ref	Ref	Ref
>1.30 to ≤3.50	0.74 (0.59, 0.93)	0.010	0.79 (0.64, 0.96)	0.022
>3.50	0.69 (0.55, 0.87)	0.002	0.69 (0.54, 0.86)	0.002
Not recorded			0.75 (0.52, 1.07)	0.109
BMI				
<25	Ref	Ref	Ref	Ref
≥25 to <30	1.21 (0.95, 1.55)	0.123	1.22 (0.97, 1.54)	0.092
≥30	1.47 (1.15, 1.88)	0.003	1.48 (1.18, 1.86)	<0.001

Variable	Primary results		Sensitivity results	
	**OR (95% CI)**	***P*-value**	**OR (95% CI)**	***P*-value**
Not recorded			2.52 (1.61, 3.94)	<0.001
Calcium				
<9.5	Ref	Ref	Ref	Ref
≥9.5	1.00 (0.82, 1.21)	0.978	0.99 (0.82, 1.20)	0.948
Not recorded			0.86 (0.61, 1.22)	0.402
Smoking				
No	Ref	Ref	Ref	Ref
Yes	1.43 (1.18, 1.73)	<0.001	1.46 (1.21, 1.75)	<0.001

Model 3: multivariable analysis for the association between smoking and osteoporosis, adjusted for age, gender, race, education, marital status, ratio of family income to poverty (PIR), body mass index (BMI), and calcium. PIR: ratio of family income to poverty; BMI: body mass index; OR: odds ratio; CI: confidence interval; Ref: reference.

Table S25 The smoking (exposure) and osteoporosis/osteoporotic fracture (outcome) GWAS summary datasets extracted from the IEU Open GWAS database

**GWAS ID**	**Year**	**Trait**	**Consortium**	**Sample size**			**Number of SNPs**	**Population**
				**Case**	**Control**	**Total**		
ukb-a-16	2017	Current tobacco smoking	Neale Lab	-	-	337,030	10,894,596	European
ukb-a-17	2017	Past tobacco smoking	Neale Lab	-	-	310,749	10,894,596	European
ukb-a-224	2017	Smoking status: Previous	Neale Lab	118,419	217,605	336,024	10,894,596	European
ukb-a-225	2017	Smoking status: Current	Neale Lab	33,928	302,096	336,024	10,894,596	European
ukb-b-223	2018	Current tobacco smoking	MRC-IEU	-	-	462,434	9,851,867	European
ukb-b-2134	2018	Past tobacco smoking	MRC-IEU	-	-	424,960	9,851,867	European
finn-b-M13_OSTEOPOROSIS	2021	Osteoporosis	-	3,203	209,575	212,778	16,380,452	European
finn-b-OSTEOPOROSIS_FRACTURE_FG	2021	Osteoporosis with pathological fracture (FG)	-	785	172,834	173,619	16,380,281	European

GWAS: genome-wide association study; SNP: single nucleotide polymorphism.

Table S26 Two-sample MR results of causal links between smoking and osteoporosis/osteoporotic fracture by using the IVW method based on the summary-level data of GWAS datasets from the IEU Open GWAS database

Exposure (Smoking)	Outcome (Osteoporosis/osteoporotic fracture)	Method	IVs	OR (95% CI)	P-value	Heterogeneity (*P*-value)		Pleiotropy (*P*-value)	
						IVW	MR-Egger	MR-PRESSO	MR-Egger
ukb-a-16	finn-b-M13_OSTEOPOROSIS	IVW	16	2.66 (0.57, 12.43)	0.213	0.567	0.613	0.566	0.235
ukb-a-17	finn-b-M13_OSTEOPOROSIS	IVW	42	1.37 (0.94, 2.01)	0.105	0.628	0.601	0.674	0.542
ukb-a-224	finn-b-M13_OSTEOPOROSIS	IVW	19	0.65 (0.11, 3.73)	0.630	0.267	0.348	0.205	0.142
ukb-a-225	finn-b-M13_OSTEOPOROSIS	IVW	15	6.51 (0.20, 210.04)	0.290	0.170	0.127	0.166	0.928
ukb-b-223	finn-b-M13_OSTEOPOROSIS	IVW	34	1.61 (0.40, 6.59)	0.505	0.069	0.073	0.079	0.311
ukb-b-2134	finn-b-M13_OSTEOPOROSIS	IVW	94	1.18 (0.88, 1.60)	0.273	0.782	0.830	0.743	0.083
ukb-a-16	finn-b-OSTEOPOROSIS_FRACTURE_FG	IVW	16	24.51 (1.11, 539.37)	0.043	0.745	0.753	0.776	0.341
ukb-a-17	finn-b-OSTEOPOROSIS_FRACTURE_FG	IVW	42	1.52 (0.71, 3.26)	0.287	0.781	0.745	0.761	0.996
ukb-a-224	finn-b-OSTEOPOROSIS_FRACTURE_FG	IVW	19	0.07 (0.00, 1.63)	0.097	0.673	0.646	0.701	0.471
ukb-a-225	finn-b-OSTEOPOROSIS_FRACTURE_FG	IVW	15	40.66 (0.05, 30808.75)	0.273	0.251	0.200	0.263	0.769
ukb-b-223	finn-b-OSTEOPOROSIS_FRACTURE_FG	IVW	34	3.95 (0.23, 67.28)	0.343	0.064	0.125	0.072	0.063
ukb-b-2134	finn-b-OSTEOPOROSIS_FRACTURE_FG	IVW	94	1.10 (0.60, 2.02)	0.756	0.998	0.999	0.998	0.032

IV: instrumental variable; OR: odds ratio; CI: confidence interval; MR: Mendelian randomization; IVW: inverse variance weighted.

Table S27 Characteristics of SNPs screened from the ukb-a-16 smoking GWAS summary dataset from the IEU Open GWAS database

SNP	Chr	EA	OA	EAF	Beta	SE	*P*-value	F-statistic
rs11210887	1	A	G	0.7003	-0.0095	0.0014	3.463E-11	44
rs3773814	3	C	A	0.1533	0.0101	0.0018	4.041E-08	30
rs11096777	4	C	T	0.1825	0.0099	0.0017	7.368E-09	33
rs1549214	5	C	T	0.6406	-0.0090	0.0014	5.623E-11	43
rs7726560	5	T	G	0.4386	0.0075	0.0013	2.199E-08	31
rs7807019	7	G	A	0.4604	0.0076	0.0013	1.027E-08	33
rs62474713	7	A	G	0.4986	-0.0087	0.0013	5.596E-11	43
rs17730481	8	A	G	0.3230	0.0086	0.0014	1.713E-09	36
rs73227362	8	C	A	0.1957	-0.0098	0.0017	3.670E-09	35
rs113382419	9	A	C	0.1121	0.0213	0.0021	3.999E-24	103
rs10891481	11	G	A	0.3826	0.0084	0.0014	5.820E-10	38
rs8033799	15	C	A	0.2114	0.0089	0.0016	3.898E-08	30
rs56113850	19	C	T	0.5777	-0.0114	0.0013	1.367E-17	73
rs4809542	20	G	C	0.0703	0.0153	0.0026	3.324E-09	35
rs6062496	20	A	G	0.5447	0.0073	0.0013	3.387E-08	30
rs9607805	22	T	C	0.7278	0.0094	0.0015	2.262E-10	40

SNP: single nucleotide polymorphism; GWAS: genome-wide association study; Chr: chromosome; EA: effect allele; OA: other allele; EAF: frequency of effect allele; SE: standard error.

Supplementary Figures

Figure S1 Triangular shape graph representing the Mendelian randomization (MR) framework. Instrumental variable (IV) assumption 1: IV is closely associated with the exposure; IV assumption 2: IV is not associated with confounders; IV assumption 3: IV can only influence the outcome through the exposure and not in other ways.

Figure S2 Scatter plot of the two-sample MR analysis outcome of smoking on the risk of osteoporotic fracture based on the summary-level data of the GWAS datasets ukb-a-16 (exposure: smoking) and finn-b-OSTEOPOROSIS_FRACTURE_FG (outcome: osteoporotic fracture) from the IEU Open GWAS database. MR: Mendelian randomization; GWAS: genome-wide association studies; SNP: single nucleotide polymorphism.

Figure S3 The forest plot was produced using each of the smoking SNPs as separate instruments based on the summary-level data of the GWAS datasets ukb-a-16 (exposure: smoking) and finn-b-OSTEOPOROSIS_FRACTURE_FG (outcome: osteoporotic fracture) from the IEU Open GWAS database. The red points show the combined causal estimate of MR-Egger and IVW methods. SNP: single nucleotide polymorphism; MR: Mendelian randomization; IVW: inverse variance weighted.

Figure S4 Leave-one-out sensitivity analyses based on the summary-level data of the GWAS datasets ukb-a-16 (exposure: smoking) and finn-b-OSTEOPOROSIS_FRACTURE_FG (outcome: osteoporotic fracture) from the IEU Open GWAS database. Each black point represents the MR method of IVW applied to estimate the causal effect of smoking on osteoporotic fracture excluding that particular variant from the analysis. The red point depicts the IVW method estimate using all SNPs. MR: Mendelian randomization; IVW: inverse variance weighted; SNP: single nucleotide polymorphism.

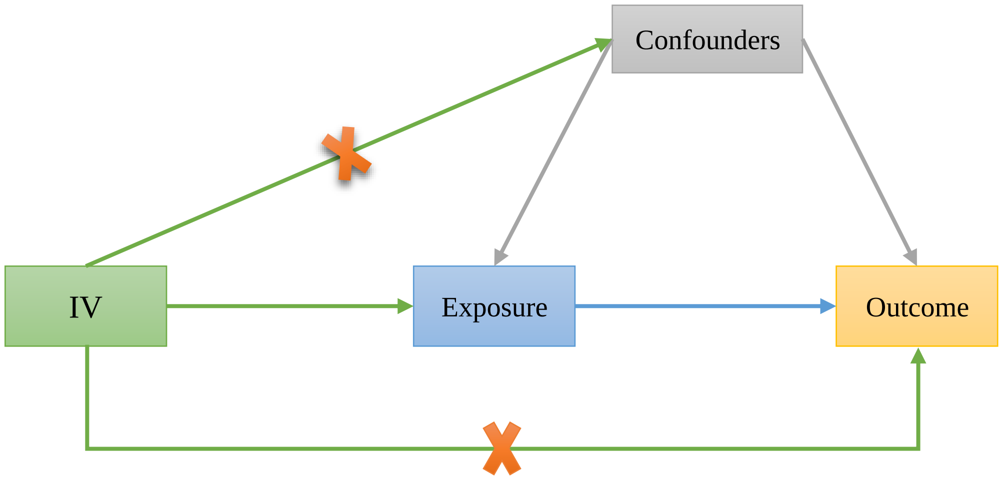
Confounders
IV
Exposure
Outcome

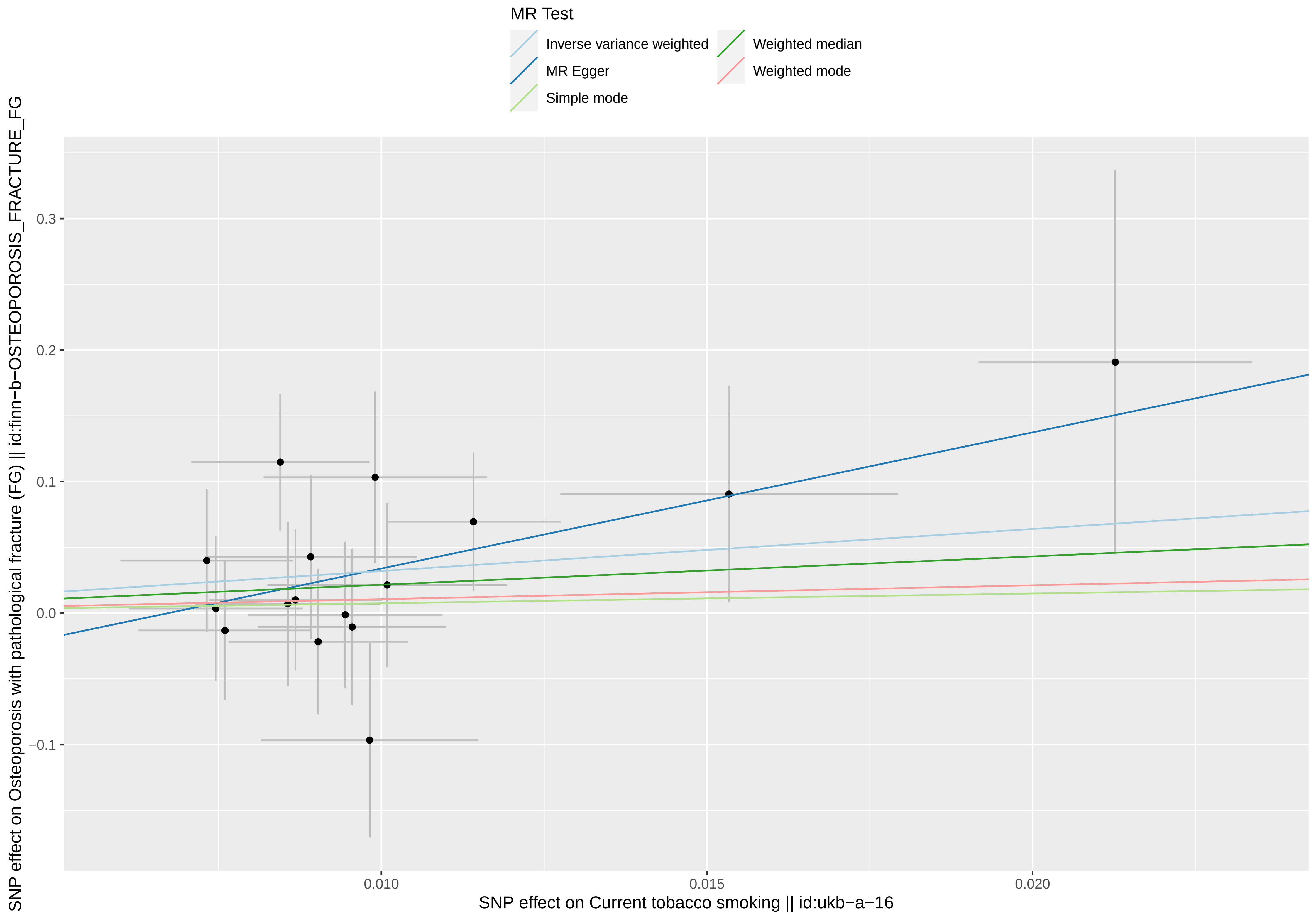
MR Test
Inverse variance weighted
MR Egger
Simple mode
Weighted median
Weighted mode
0.3
0.2
0.1
0.0
−0.1
0.010
0.015
0.020
SNP effect on Osteoporosis with pathological fracture (FG) || id:finn−b−OSTEOPOROSIS_FRACTURE_FG
SNP effect on Current tobacco smoking || id:ukb−a−16

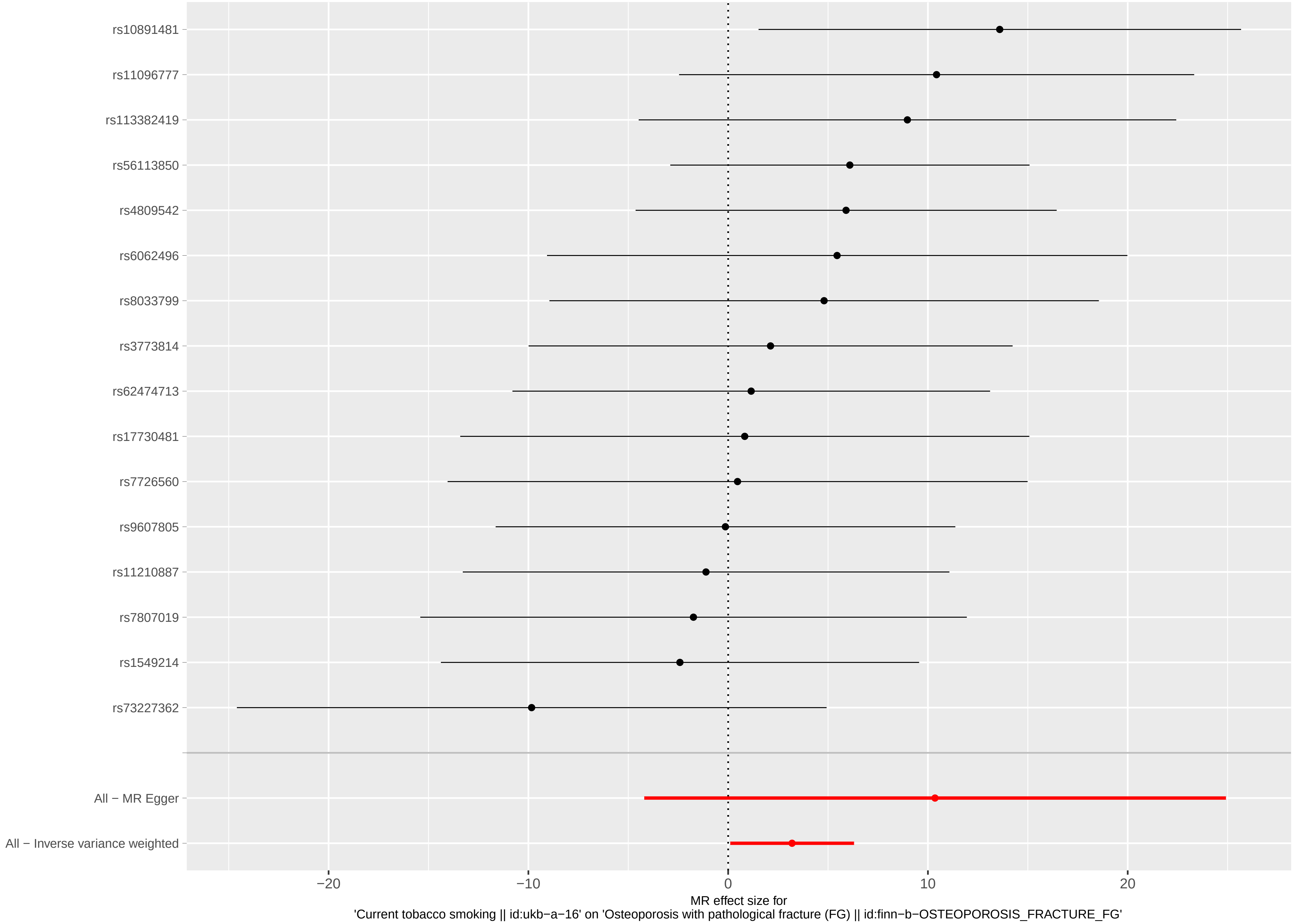
rs10891481
rs11096777
rs113382419
rs56113850
rs4809542
rs6062496
rs8033799
rs3773814
rs62474713
rs17730481
rs7726560
rs9607805
rs11210887
rs7807019
rs1549214
rs73227362
All − MR Egger
All − Inverse variance weighted
−20
−10
0
10
20
MR effect size for
'Current tobacco smoking || id:ukb−a−16' on 'Osteoporosis with pathological fracture (FG) || id:finn−b−OSTEOPOROSIS_FRACTURE_FG'

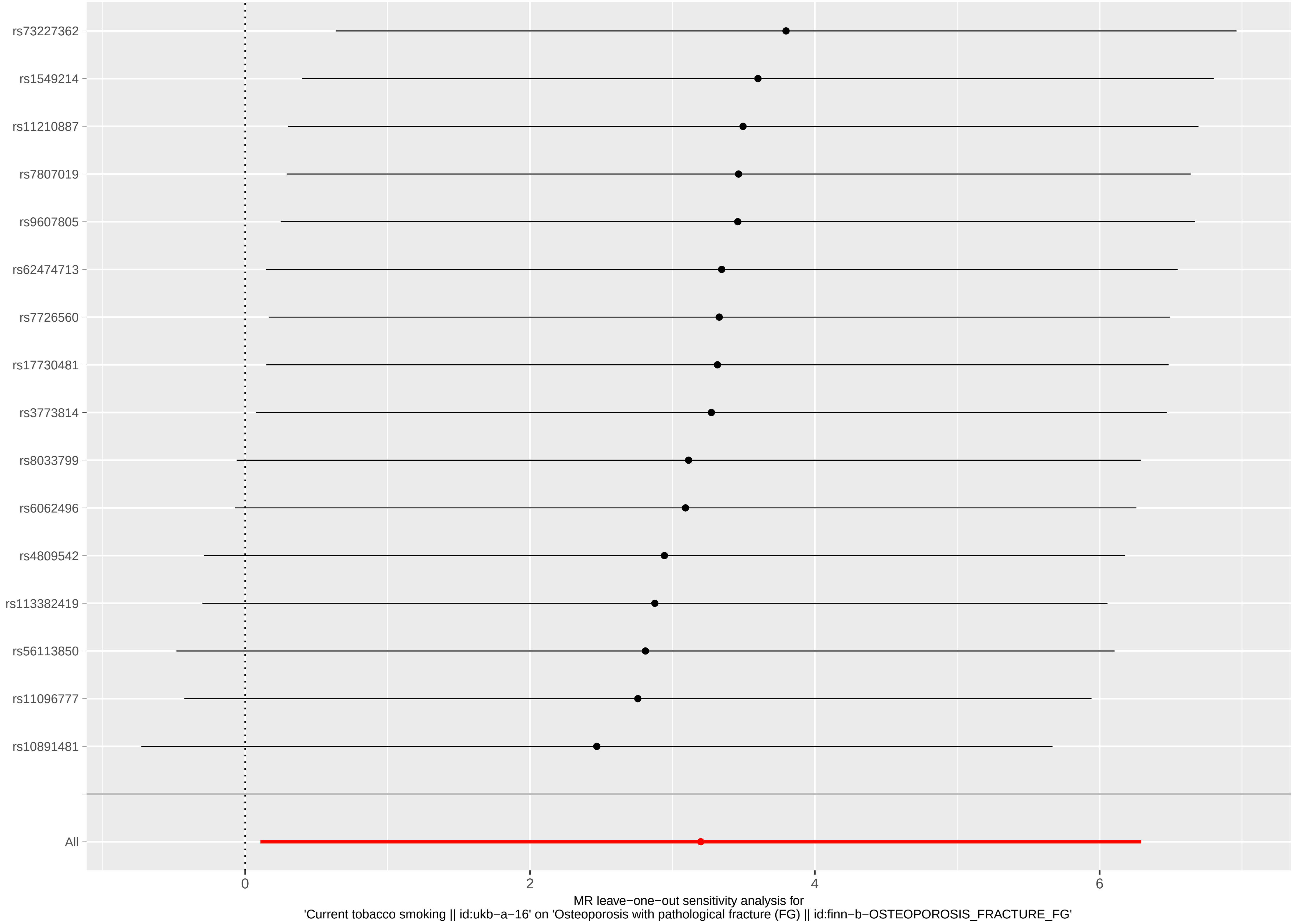
rs73227362
rs1549214
rs11210887
rs7807019
rs9607805
rs62474713
rs7726560
rs17730481
rs3773814
rs8033799
rs6062496
rs4809542
rs113382419
rs56113850
rs11096777
rs10891481
All
0
2
4
6
MR leave−one−out sensitivity analysis for
'Current tobacco smoking || id:ukb−a−16' on 'Osteoporosis with pathological fracture (FG) || id:finn−b−OSTEOPOROSIS_FRACTURE_FG'

STROBE Statement—checklist of items that should be included in reports of observational studies

	Item No	Recommendation	Page No
**Title and abstract**	1	(*a*) Indicate the study's design with a commonly used term in the title or the abstract	1
		(*b*) Provide in the abstract an informative and balanced summary of what was done and what was found	2-3
**Introduction**			
Background/rationale	2	Explain the scientific background and rationale for the investigation being reported	3-5
Objectives	3	State specific objectives, including any prespecified hypotheses	4
**Methods**			
Study design	4	Present key elements of study design early in the paper	5-6
Setting	5	Describe the setting, locations, and relevant dates, including periods of recruitment, exposure, follow-up, and data collection	5-6
Participants	6	(*a*) *Cohort study*—Give the eligibility criteria, and the sources and methods of selection of participants. Describe methods of follow-up	N/A
		Case-control study—Give the eligibility criteria, and the sources and methods of case ascertainment and control selection. Give the rationale for the choice of cases and controls	N/A
		Cross-sectional study—Give the eligibility criteria, and the sources and methods of selection of participants	5
		(*b*) *Cohort study*—For matched studies, give matching criteria and number of exposed and unexposed	N/A
		Case-control study—For matched studies, give matching criteria and the number of controls per case	N/A
Variables	7	Clearly define all outcomes, exposures, predictors, potential confounders, and effect modifiers. Give diagnostic criteria, if applicable	5-6
Data sources/ measurement	8*	For each variable of interest, give sources of data and details of methods of assessment (measurement). Describe comparability of assessment methods if there is more than one group	5-7
Bias	9	Describe any efforts to address potential sources of bias	5-7
Study size	10	Explain how the study size was arrived at	5-7
Quantitative variables	11	Explain how quantitative variables were handled in the analyses. If applicable, describe which groupings were chosen and why	5-7
Statistical methods	12	(*a*) Describe all statistical methods, including those used to control for confounding	7
		(*b*) Describe any methods used to examine subgroups and interactions	7
		(*c*) Explain how missing data were addressed	7
		(*d*) *Cohort study*—If applicable, explain how loss to follow-up was addressed	N/A
		Case-control study—If applicable, explain how matching of cases and controls was addressed	N/A
		Cross-sectional study—If applicable, describe analytical methods taking account of sampling strategy	7
		(*e*) Describe any sensitivity analyses	

Continued on next page

**Results**			
Participants	13*	(a) Report numbers of individuals at each stage of study—eg numbers potentially eligible, examined for eligibility, confirmed eligible, included in the study, completing follow-up, and analysed	9-12
		(b) Give reasons for non-participation at each stage	N/A
		(c) Consider use of a flow diagram	N/A
Descriptive data	14*	(a) Give characteristics of study participants (eg demographic, clinical, social) and information on exposures and potential confounders	9-12
		(b) Indicate number of participants with missing data for each variable of interest	10
		(c) *Cohort study*—Summarise follow-up time (eg, average and total amount)	N/A
Outcome data	15*	*Cohort study*—Report numbers of outcome events or summary measures over time	N/A
		Case-control study—Report numbers in each exposure category, or summary measures of exposure	N/A
		Cross-sectional study—Report numbers of outcome events or summary measures	10,11
Main results	16	(*a*) Give unadjusted estimates and, if applicable, confounder-adjusted estimates and their precision (eg, 95% confidence interval). Make clear which confounders were adjusted for and why they were included	9-12
		(*b*) Report category boundaries when continuous variables were categorized	9-12
		(*c*) If relevant, consider translating estimates of relative risk into absolute risk for a meaningful time period	9-12
Other analyses	17	Report other analyses done—eg analyses of subgroups and interactions, and sensitivity analyses	9-12
**Discussion**			
Key results	18	Summarise key results with reference to study objectives	14-17
Limitations	19	Discuss limitations of the study, taking into account sources of potential bias or imprecision. Discuss both direction and magnitude of any potential bias	17
Interpretation	20	Give a cautious overall interpretation of results considering objectives, limitations, multiplicity of analyses, results from similar studies, and other relevant evidence	17
Generalisability	21	Discuss the generalisability (external validity) of the study results	17
**Other information**			
Funding	22	Give the source of funding and the role of the funders for the present study and, if applicable, for the original study on which the present article is based	N/A

*Give information separately for cases and controls in case-control studies and, if applicable, for exposed and unexposed groups in cohort and cross-sectional studies.

Note: An Explanation and Elaboration article discusses each checklist item and gives methodological background and published examples of transparent reporting. The STROBE checklist is best used in conjunction with this article (freely available on the Web sites of PLoS Medicine at http://www.plosmedicine.org/, Annals of Internal Medicine at http://www.annals.org/, and Epidemiology at http://www.epidem.com/). Information on the STROBE Initiative is available at www.strobe-statement.org.

STROBE-MR checklist of recommended items to address in reports of Mendelian randomization studies[1 2]

Item No.	Section		Checklist item	Page No.	Relevant text from manuscript
1	**TITLE and ABSTRACT**		Indicate Mendelian randomization (MR) as the study's design in the title and/or the abstract if that is a main purpose of the study	1	Two-sample Mendelian randomization
	**INTRODUCTION**				
2	**Background**		Explain the scientific background and rationale for the reported study. What is the exposure? Is a potential causal relationship between exposure and outcome plausible? Justify why MR is a helpful method to address the study question	4-5	The last paragraph of the Introduction section
3	**Objectives**		State specific objectives clearly, including pre-specified causal hypotheses (if any). State that MR is a method that, under specific assumptions, intends to estimate causal effects	4-5	The last paragraph of the Introduction section
	**METHODS**				
4	**Study design and data sources**		Present key elements of the study design early in the article. Consider including a table listing sources of data for all phases of the study. For each data source contributing to the analysis, describe the following:		
		a)	Setting: Describe the study design and the underlying population, if possible. Describe the setting, locations, and relevant dates, including periods of recruitment, exposure, follow-up, and data collection, when available.	7	Study design
		b)	Participants: Give the eligibility criteria, and the sources and methods of selection of participants. Report the sample size, and whether any power or sample size calculations were carried out prior to the main analysis	8	Data sources
		c)	Describe measurement, quality control and selection of genetic variants	8-9	IV selection
		d)	For each exposure, outcome, and other relevant variables, describe methods of assessment and diagnostic criteria for diseases	8	Data sources
		e)	Provide details of ethics committee approval and participant informed consent, if relevant	18	Declaration of ethics approval and consent to participate
5	**Assumptions**		Explicitly state the three core IV assumptions for the main analysis (relevance, independence and exclusion restriction) as well assumptions for any additional or sensitivity analysis	7	Study design
6	**Statistical methods: main analysis**		Describe statistical methods and statistics used		

		a)	Describe how quantitative variables were handled in the analyses (i.e., scale, units, model)	9	Statistical analyses for MR
		b)	Describe how genetic variants were handled in the analyses and, if applicable, how their weights were selected	9	Statistical analyses for MR
		c)	Describe the MR estimator (e.g. two-stage least squares, Wald ratio) and related statistics. Detail the included covariates and, in case of two-sample MR, whether the same covariate set was used for adjustment in the two samples	9	Statistical analyses for MR
		d)	Explain how missing data were addressed	9	Statistical analyses for MR
		e)	If applicable, indicate how multiple testing was addressed	9	Statistical analyses for MR
7	**Assessment of assumptions**		Describe any methods or prior knowledge used to assess the assumptions or justify their validity	9	Statistical analyses for MR
8	**Sensitivity analyses and additional analyses**		Describe any sensitivity analyses or additional analyses performed (e.g. comparison of effect estimates from different approaches, independent replication, bias analytic techniques, validation of instruments, simulations)	9	Statistical analyses for MR
9	**Software and pre-registration**				
		a)	Name statistical software and package(s), including version and settings used	9	R packages of "TwoSampleMR" and “MR-PRESSO”
		b)	State whether the study protocol and details were pre-registered (as well as when and where)	N/A	
	**RESULTS**				
10	**Descriptive data**				
		a)	Report the numbers of individuals at each stage of included studies and reasons for exclusion. Consider use of a flow diagram	N/A	
		b)	Report summary statistics for phenotypic exposure(s), outcome(s), and other relevant variables (e.g. means, SDs, proportions)	12-13	Two-sample MR analyses
		c)	If the data sources include meta-analyses of previous studies, provide the assessments of heterogeneity across these studies	N/A	
		d)	For two-sample MR:<br>i. Provide justification of the similarity of the genetic variant-exposure associations between the exposure and outcome samples	12-13	Two-sample MR analyses

			ii. Provide information on the number of individuals who overlap between the exposure and outcome studies	12-13	Two-sample MR analyses
11	**Main results**				
		a)	Report the associations between genetic variant and exposure, and between genetic variant and outcome, preferably on an interpretable scale	12-14	Two-sample MR analyses
		b)	Report MR estimates of the relationship between exposure and outcome, and the measures of uncertainty from the MR analysis, on an interpretable scale, such as odds ratio or relative risk per SD difference	12-14	Two-sample MR analyses
		c)	If relevant, consider translating estimates of relative risk into absolute risk for a meaningful time period	N/A	
		d)	Consider plots to visualize results (e.g. forest plot, scatterplot of associations between genetic variants and outcome versus between genetic variants and exposure)	12-14	Two-sample MR analyses
12	**Assessment of assumptions**				
		a)	Report the assessment of the validity of the assumptions	13	Two-sample MR analyses
		b)	Report any additional statistics (e.g., assessments of heterogeneity across genetic variants, such as I^2, Q statistic or E-value)	13	Two-sample MR analyses
13	**Sensitivity analyses and additional analyses**				
		a)	Report any sensitivity analyses to assess the robustness of the main results to violations of the assumptions	13	Two-sample MR analyses
		b)	Report results from other sensitivity analyses or additional analyses	13	Two-sample MR analyses
		c)	Report any assessment of direction of causal relationship (e.g., bidirectional MR)	N/A	
		d)	When relevant, report and compare with estimates from non-MR analyses	N/A	
		e)	Consider additional plots to visualize results (e.g., leave-one-out analyses)	N/A	
	**DISCUSSION**				
14	**Key results**		Summarize key results with reference to study objectives	16	The sixth paragraph of the Discussion section
15	**Limitations**		Discuss limitations of the study, taking into account the validity of the IV assumptions, other sources of potential bias, and imprecision. Discuss both direction and magnitude of any potential bias and any efforts to address them	17	The eighth paragraph of the Discussion section

16	**Interpretation**			
		a) Meaning: Give a cautious overall interpretation of results in the context of their limitations and in comparison with other studies	17	The eighth paragraph of the Discussion section
		b) Mechanism: Discuss underlying biological mechanisms that could drive a potential causal relationship between the investigated exposure and the outcome, and whether the gene-environment equivalence assumption is reasonable. Use causal language carefully, clarifying that IV estimates may provide causal effects only under certain assumptions	15	The fourth paragraph of the Discussion section
		c) Clinical relevance: Discuss whether the results have clinical or public policy relevance, and to what extent they inform effect sizes of possible interventions	17	The eighth paragraph of the Discussion section
17	**Generalizability**	Discuss the generalizability of the study results (a) to other populations, (b) across other exposure periods/timings, and (c) across other levels of exposure	17	The eighth paragraph of the Discussion section
	**OTHER INFORMATION**			
18	**Funding**	Describe sources of funding and the role of funders in the present study and, if applicable, sources of funding for the databases and original study or studies on which the present study is based	N/A	
19	**Data and data sharing**	Provide the data used to perform all analyses or report where and how the data can be accessed, and reference these sources in the article. Provide the statistical code needed to reproduce the results in the article, or report whether the code is publicly accessible and if so, where	19	Availability of data and materials
20	**Conflicts of Interest**	All authors should declare all potential conflicts of interest	19	Competing interests

This checklist is copyrighted by the Equator Network under the Creative Commons Attribution 3.0 Unported (CC BY 3.0) license.

1. Skrivankova VW, Richmond RC, Woolf BAR, Yarmolinsky J, Davies NM, Swanson SA, et al. Strengthening the Reporting of Observational Studies in Epidemiology using Mendelian Randomization (STROBE-MR) Statement. JAMA. 2021;under review.

2. Skrivankova VW, Richmond RC, Woolf BAR, Davies NM, Swanson SA, VanderWeele TJ, et al. Strengthening the Reporting of Observational Studies in Epidemiology using Mendelian Randomisation (STROBE-MR): Explanation and Elaboration. BMJ. 2021;375:n2233.

©2024 Fang M. et al.
